# High burden of depressive symptoms among sexual and gender diverse persons in Brazil: The role of discrimination, internalized homonegativity, and race

**DOI:** 10.1371/journal.pgph.0007276

**Published:** 2026-09-28

**Authors:** Caroline Guimarães, Paula Mendes Luz, Carolina Coutinho, Emilia M. Jalil, Cristina M. Jalil, Beatriz Grinsztejn, Valdilea Gonçalves Veloso, Lucilene Araújo Freitas, Thiago Silva Torres

**Affiliations:** Laboratório de Pesquisa Clínica em IST, hepatites e AIDS (LapClin-AIDS), Instituto Nacional de Infectologia Evandro Chagas (INI), Fundação Oswaldo Cruz (Fiocruz), Rio de Janeiro, Brazil; The University of Sydney, AUSTRALIA

## Abstract

Sexual and gender diverse (SGD) populations have historically faced restricted rights and systematic discrimination for deviating from the established hetero-cis-normative norms. This long-standing marginalization has a profound negative impact on health outcomes, including a heightened risk of developing mental health issues, such as depression. Understanding the health-disease process among SGD populations requires recognizing that discriminatory experiences, such as homophobia and transphobia, are not unidimensional, but operate synergistically with layered forms of discrimination, such as racism. We assessed depressive symptoms using the Patient Health Questionnaire-9 (PHQ-9) among SGD persons in Brazil using race as a stratification variable. We also evaluated the role of discrimination and internalized homonegativity on depressive symptoms, while adjusting for covariates. This was a cross-sectional online survey recruiting SGD persons aged 18 years or older, living in Brazil, through dating apps and social media. Overall, 6220 participants were included, 3749 (60.3%) White, 1685 (27.1%) *Pardo* and 786 (12.6%) Black. Mean scores of depressive symptoms was 8.8 (SD7.1), higher among Black (9.6; SD 7.5) and *Pardo* (9.3; SD 7.2) than White (8.4; SD7.0) (p < 0.0001). Considering a PHQ-9 cut-off of ≥10 points, more Black and *Pardo* participants reported depressive symptoms than White participants (38.5%, 38.1%; 32.3%, respectively). In multivariable linear regression models, higher discrimination score and younger age were associated with higher scores of depressive symptoms for all races. Additionally, internalized homonegativity and lower income were associated with higher depressive symptoms scores for *Pardo* and White, but not for Black participants. To effectively address vulnerability to depression among SGD populations, it is essential to establish approaches that consider the intersections between different forms of oppression. This includes policies that tackle racism, homophobia, and transphobia, as well as investment in accessible mental health services.

## Introduction

Depression is a common mental disorder, affecting an estimated 5.7% of adults globally [1]. Despite the availability of effective and recognized treatments, more than 75% of people with depression do not have access to care in low- and middle-income countries, mainly due to insufficient investments in mental health care and the stigma surrounding mental disorders [2]. As a multifactorial phenomenon, depression is not understood as an essentially individual condition, but rather as the result of the interaction between genetic, psychological, and environmental factors, which can be influenced by stressful events such as loss of loved ones, violence and discrimination [1].

Stigma, prejudice, and discrimination are interconnected social phenomena that produce suffering and act as social determinants of health [3]. Stigma is the association of a person with a stereotype or an attribute that society deems undesirable, reducing them to a single, discredited trait [4,5]. Prejudice is defined as a hostile attitude towards a person simply because they belong to a certain group resulting from the assumption that they possess the negative characteristics that are attributed to the group [5]. Both stigma and prejudice involve categorization, labelling, stereotyping and social rejection, resulting in discrimination and exclusion [6]. Associated with stigma and prejudice, discrimination refers to the practice or behavior of unfair or unequal treatment based on personal characteristics or perceived identities, such as race, ethnicity, gender, sexual orientation, socioeconomic status, among others [7]. This differential treatment can manifest itself in a variety of ways, including marginalization, exclusion, harassment and violence.

Discrimination can occur at the individual level, being reproduced in interpersonal interactions, and at structural levels, in policies, laws and institutions that perpetuate inequalities and injustices that affect certain groups [3]. Developed by Kimberlé Crenshaw in the late 1980s, the term intersectionality initially considered the interaction between race and gender in the experiences of Black women, and was later expanded to other populations [8,9]. Considering the impossibility of analyzing in isolation the factors that affect individuals’ lives, intersectionality provides visibility to the inequalities that impact the existence of certain marginalized groups who are systematically exposed to discriminatory situations, and it allows for the development of responses that address their specific needs [9].

Since the 1990s, a series of studies have provided evidence for the development of a theoretical model for the understanding of mental health conditions as experienced by people who suffer constant oppression [10]. The minority stress theory, developed by the epidemiologist Ilan H. Meyer, was initially conceptualized for situations experienced by gay men in the U.S. Over time, however, the term has evolved to more broadly include various minority groups, such as other sexual and gender diverse (SGD) persons, people with disabilities, Indigenous people and Black people. The theory represented an important advancement in understanding the mental health aspects of stigmatized groups and suggests that the more minority characteristics a person possesses, the greater the damage to their mental health, implying an intersectional aspect.

Historically, SGD populations have had their rights restricted and have been victims of discrimination for diverging from the established heterosexual norm. This constant exposure to discrimination may have a negative impact on the physical and mental health of these populations [11]. To understand the health–illness process among SGD populations, it is essential to recognize that different forms of discrimination affect this group, such as lesbophobia, homophobia, biphobia, and transphobia. These forms of discrimination do not occur in isolation but are reinforced and intersect with other forms of prejudice, such as racism, sexism, and misogyny [12]. Among Black SGD persons, various intersections can be observed, such as an increased risk of violence due to skin color, gender identity, and sexual orientation, which directly impacts their psychological well-being [13]. In Brazil, systemic racism has persisted for centuries, resulting in social vulnerability and worse health outcomes for Black, *Pardo*, and Indigenous populations [14]. In Brazil, as an effect of racism, a large part of the Black population live in constant suffering due to precarious living conditions and the lack of prospects, which produces stress and anguish and may consequently lead to mental disorders including depression [15]. A systematic review of studies conducted in Brazil found a higher prevalence of depressive symptoms among Black women compared to White women, and among nonwhite older adults compared to their White counterparts [16].

Both discrimination and internalized homonegativity may constitute significant stressors in the lives of SGD persons, potentially exerting a range of adverse effects on their mental health. Frequent exposure to prejudice leads to the internalization of negative messages about one’s own sexual and gender identity, which can result in low self-esteem and feelings of self-hatred, causing these individuals to withdraw from their communities and avoid social situations that could expose them to the risk of discrimination [17]. This constant worry about the possibility of being the target of prejudice or violence can contribute to feelings of hopelessness, sadness and isolation, increasing the risk of depression or other mental health disorders [18]. This study aimed to examine the associations of discrimination and internalized homonegativity with depressive symptoms among SGD persons in Brazil, with analyses stratified by race.

## Materials and methods

### Study design and questionnaire

This was a cross-sectional online survey enrolling a convenience sample recruited through dating apps (Grindr, Hornet and Scruff, three of the most used dating apps by SGD persons in Brazil) and social media (Facebook and Instagram) from November 07 2021 to January 29 2022. Invitations to voluntarily participate in the survey were sent via direct messages on dating apps and boosted posts on social media. Details of study design and results from the primary analysis are described elsewhere [7]. Inclusion criteria were: a) self-identifying as a person of a SGD identity, b) age 18 years and older, and c) living in Brazil. Exclusion criteria were: a) failure to provide informed consent; b) identifying as a cisgender woman; c) identifying as cisgender men and reporting never having had sexual relations with other men; d) answering any of the attention questions incorrectly; and e) not reaching the end of the questionnaire. For this analysis, we additionally excluded those who: a) identified as Asian (n = 103) and Indigenous (n = 73) due to the small sample size, b) had missing information on race (n = 129), and c) did not complete the Patient Health Questionnaire-9 (PHQ-9) (Fig 1). The study questionnaire included questions about sociodemographic characteristics, substance use, binge drinking, HIV status, discrimination, internalized homonegativity, and depressive symptoms. The questionnaire was in Portuguese, and a non-response option was available for all items.

**Fig 1:**
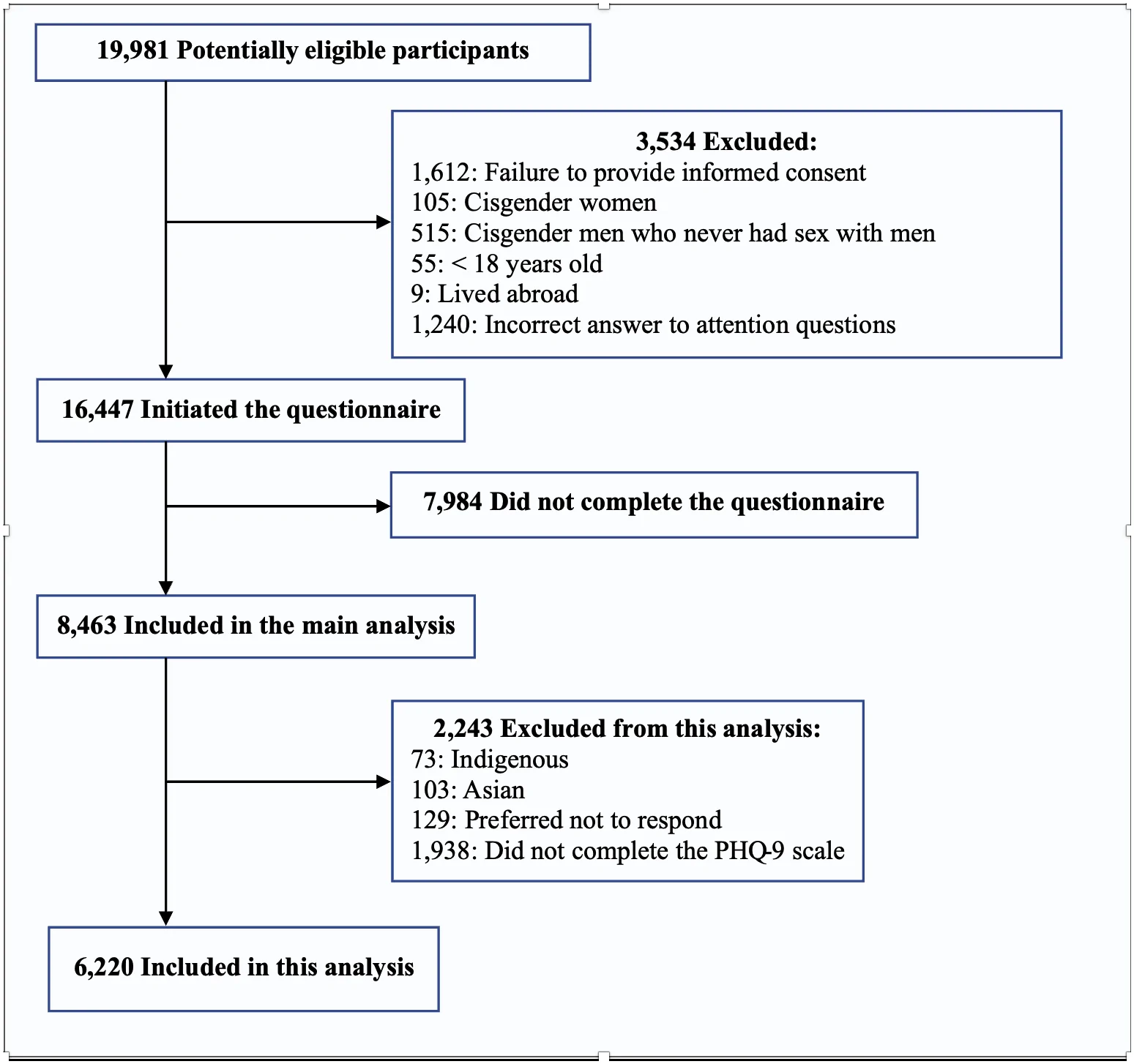
Study flowchart, Brazil, November 2021 to January 2022.

### Ethics statement

The study was approved by the Institutional Review Board at Instituto Nacional de Infectologia Evandro Chagas, Fundação Oswaldo Cruz (INI-Fiocruz; #CAAE 01777918.0.0000.5262). All study participants provided digital informed consent before survey initiation. No compensation was provided.

### Outcome

The PHQ-9, validated in Brazilian Portuguese [19], is a brief instrument widely used to screen individuals with severe depressive symptoms [20]. The instrument contains nine questions evaluating each symptom of a major depressive episode present in the Diagnostic and Statistical Manual of Mental Disorders (DSM-IV), and a tenth question that assesses the impact of these symptoms on daily functioning. The frequency of each symptom in the last two weeks was assessed on a *Likert* scale of 0–3 points corresponding to the answers “not at all” (0), “several days” (1), “more than half the days” (2) and “almost every day” (3). PHQ-9 scores were further classified into no symptoms (0–4), mild symptoms (5–9), moderate symptoms (10–14), moderate to severe symptoms (15–19), and severe symptoms (20–27). Nevertheless, high scores are not diagnostic; a definitive diagnosis can only be established through consultations with mental health professionals.

### Main exposures

#### Discrimination.

The Explicit Discrimination Scale (EDS) instrument, first developed with 18-items, assesses the occurrence of explicit discrimination in day-to-day situations that are applicable to the Brazilian context [21]. This instrument was the first to assess explicit discrimination outside the context of high-income countries. To evaluate discrimination, it is essential to consider cultural, historical, and social contexts of a given society. In Brazil, social and racial conflicts often occur in a more subtle manner due to the myth of racial democracy, which has historically hidden inequalities [22]. The EDS is suitable for our analysis for three reasons: it addresses a wide range of situations that tend to be discriminatory; it allows participants to associate their experiences of discrimination with one or more factors simultaneously; and it considers that discrimination and the perception of a discriminatory event are distinct yet interrelated phenomena [23].

We used the abbreviated 8-item EDS which assesses perceived discrimination in 8 different circumstances. It has a robust capacity to address intersections between the different axes of inequality that interact and are experienced simultaneously [24]. Prior work has shown that the abbreviated EDS version was suitable for analysis of comparable experiences of discrimination among groups defined by sex, race, and education [23].

The abbreviated EDS contains the following items related to differential treatment: (1) treated disrespectfully in public places; (2) treated as unintelligent at school or college; (3) treated as unintelligent in the workplace; (4) unfair evaluation in workplace; (5) called names the person doesn't like; (6) excluded or left aside by friends at school; (7) excluded or left aside by coworkers; and (8) excluded or left aside by people in the neighborhood. Response options were (coding): never (0), occasionally (1), often (2), and always (3). Participants who reported a specific experience of differential treatment were asked whether they perceived it as discrimination (yes or no). If yes, responses to the question about differential treatment, across the 8-items, were summed to yield the participant’s discrimination score, which ranged from 0 to 24. Higher scores indicated more discriminatory experiences. Participants also answered about the main and additional reasons for each reported discriminatory experience, with 12 pre-defined response options (race, social class, sexual orientation, gender, physical disability, age, residence location, accent, clothing, overweight, disease, appearance, political orientation, religion, and other - which the participant needed to specify).

#### Internalized homonegativity.

Internalized homonegativity is a term used to describe negative feelings that a person may have towards themselves because of their own sexual orientation. This phenomenon occurs when individuals introject prejudices, stigmas, and negative social norms about their way of experiencing sexuality [10].

The Reactions to Homosexuality Scale (RHS) assesses three main dimensions: a) social comfort with lesbian, gay, bisexual, transgender, queer, intersex, asexual (or agender/aromantic), pansexual and non-binary (LGBTQIAPN+) persons, with two items measuring well-being in social situations with LGBTQIAPN+ persons and places such as LGBTQIAPN+ bars; b) public identification as LGBTQIAPN+ person and personal comfort with a LGBTQIAPN+ identity, with two items measuring comfort with talking about homosexuality in public or being seen with an openly LGBTQIAPN+ person; and c) personal comfort with LGBTQIAPN+ identity, with three items assessing comfort with one’s own sexuality and the belief that homosexuality is as natural as heterosexuality [25]. The score is calculated by summing all items (ranging from 0 to 42). Higher scores indicate greater internalized homonegativity. This instrument was validated in a sample of gay, bisexual and other men who have sex with men [26] and used in other studies with SGD populations from Brazil [27–29].

### Co-variables

Age was described in median and interquartile range (IQR) and stratified as 18–24, 25–35, > 35 years, following previous studies [7,28]. Race was categorized according to the definition of the Brazilian Institute of Geography and Statistics (IBGE): a) Asian, for people who identify themselves as Asian or of Asian descent, such as Japanese, Chinese, Koreans and others; b) Black, for people who identify themselves as Black or of African descent; c) Indigenous, those who identify themselves as Indigenous, belonging to one or more of the various indigenous ethnic groups in Brazil; d) *Pardo*, people who declare themselves as mixed race or multiracial, in a combination of Black, Indigenous or White ancestry; and e) White, people who identify themselves as white or of European ancestry. *Pardo* is a specific race/color category used in Brazil that is characterized by a skin tone intermediate between black and white and by the consequent greater degree of mobility between these two chromatic extremes [30]. Gender was categorized in cisgender man, transgender man, transgender woman, *travesti* (in Latin America refers to a person who was assigned male at birth but identifies and expresses a feminine gender identity) and non-binary person. Sexual orientation refers to the internal experience related to affective-sexual attraction to another person [31]. This variable was collected as: a) asexual (absence of affective-sexual attraction to people of any gender); b) bisexual (affective-sexual attraction to people of any gender); c) gay or homosexual (affective-sexual attraction to people of the same gender as the one with which one identifies); d) heterosexual (affective-sexual attraction to people of a different gender to the one with which they identify); e) pansexual (affective-sexual attraction to people regardless of gender), and f) other. Education was categorized into elementary or less, secondary and tertiary or higher. Monthly family income was inquired in relation to the monthly minimum wage (MW), which was R$ 1,212 at the time of the study and was equivalent to US$ 212. This variable was categorized into low (up to 2 MW), middle (more than 2–6 MW), and high (more than 6 MW). The Brazilian state of residence was recorded and categorized according to the country’s administrative regions: North (seven states), Northeast (nine states), Midwest (three states and the Federal District), Southeast (four states) and South (three states). Definition of binge drinking was consuming five or more standard drinks (1 can of beer [340 mL] or 1 glass of wine [140 mL] or 1 shot of distilled alcohol [40 mL of ex. cachaça, vodka, whisky]) on a single occasion in the past year [32]. Substance use included any illicit substance used in the past 6 months, such as marijuana, hash, cocaine, crack, amphetamines, lysergic acid (LSD) and ketamine (“special K”). Participants were asked whether they had ever been tested for HIV and, if so, the result of their most recent test (negative, positive, or unknown).

### Statistical analysis

First, we described the sample overall and according to self-identified race (Black, *Pardo* or White). We compared sociodemographic characteristics, binge drinking, substance use, HIV status, discrimination, internalized homonegativity, and symptoms of depression (PHQ-9 mean scores and standard deviations [SD] and categories) according to participants’ race using the Chi-square test for categorical variables and ANOVA and Kruskal-Wallis tests for continuous variables. Then, we described the proportion of individuals reporting “not at all” (0), “several days” (1), “more than half the days” (2) and “almost every day” per item of the PHQ-9 scale, according to race. We further describe the reported reasons for discrimination (either main or additional) across the 8 items of EDS stratified by race.

We used linear regression models stratified by race to assess if the exposures (discrimination and internalized homonegativity) and other covariables (sociodemographic, binge drinking, substance use, and HIV status) were associated with the outcome. Models were stratified by race due to differential experiences of discrimination observed in prior work by our group, which found that Black SGD experienced more discrimination compared to *Pardo* and White participants, with a unique contribution of racism to those disparities [7,33]. Our modeling approach included multiple sequential models, adding variables in a specific sequence to see how the effect size of the main exposures changed after including additional variables or confounders. We started investigating the association of discrimination with depression, followed by the association of internalized homonegativity with depression. Then, we assessed the association of discrimination and internalized homonegativity with depression. Lastly, we added other covariates to this model. We evaluated the outcome in the model as a continuous variable, with higher scores of PHQ-9 indicating higher symptoms of depression. Though the PHQ-9 cut-off score of 10 or more has been used to estimate prevalence of depressive symptoms, evidence suggests that this cut-off substantially overestimates the prevalence and should be used with caution [34]. Due to its high sensitivity, the use of PHQ-9 score as a continuous variable proved to be more suitable than with the cut-off for screening major depressive symptoms in a Brazilian study [19]. Scale scores (outcome and factors) were standardized to enable comparisons of the estimated model coefficients.

## Results

Overall, 6220 participants were included in this analysis, 3749 (60.3%) self-identified as White, 1685 (27.1%) as *Pardo*, and 786 (12.6%) as Black (Table 1). Most were recruited through dating apps Grindr, Hornet and Scruff (90.6%). Median age was 36 years (interquartile range [IQR]: 29–44); most self-identified as cisgender men who have sex with men (MSM) (97.8%), as gay (84.1%), completed tertiary education (75.4%), had low or middle income (66.5%), were from the Southeast Brazil (69.1%), lived in a state capital (73.4%), and reported HIV negative status (67.9%). Substance use and binge drinking were reported by 35.7% and 30.0%, respectively. Compared to White participants, Black participants were younger, and a higher proportion reported: sexual orientation other than gay, lower education and income, living in the Northeast region, in a state capital, and binge drinking (all p < 0.001).

**Table 1:** Sociodemographic characteristics, substance use, HIV status, internalized homonegativity (measured by Reactions to Homosexuality Scale), discrimination (measured by Explicit Discrimination Scale) and depressive symptoms (measured by Patient Health Questionnaire, 9 items) overall and stratified by race.

	Total	White	*Pardo*	Black	p-value
	N = 6220	N = 3749 (60.3%)	N = 1685 (21.3%)	N = 786 (12.6%)	
**Recruitment**					< 0.001
Grindr	1313 (21.1)	742 (19.8)	387 (23.0)	184 (23.4)	
Hornet	2386 (38.4)	1563 (41.7)	578 (34.3)	245 (31.2)	
Scruff	1939 (31.2)	1117 (29.8)	545 (32.3)	277 (35.2)	
Facebook/Instagram	582 (9.4)	327 (8.7)	175 (10.4)	80 (10.2)	
**Age (years)**					
Median (IQR)	36 (29-44)	37 (30-45)	35 (29-42)	33 (27-39)	< 0.001
18-24	543 (8.7)	292 (7.8)	160 (9.5)	91 (11.6)	< 0.001
25-35	2514 (40.4)	1406 (37.5)	722 (42.8)	386 (49.1)	
>35	3163 (50.9)	2051 (54.7)	803 (47.7)	309 (39.3)	
**Gender**					0.005
Cisgender man	6081 (97.8)	3670 (97.9)	1653 (98.1)	758 (96.4)	
Transgender man	12 (0.2)	4 (0.1)	5 (0.3)	3 (0.4)	
Transgender woman	12 (0.2)	6 (0.2)	2 (0.1)	4 (0.5)	
*Travesti*	5 (0.1)	1 (0)	4 (0.2)	0 (0)	
Non-binary person	110 (1.8)	68 (1.8)	21 (1.2)	21 (2.7)	
**Sexual orientation**					< 0.001
Gay	5226 (84.1)	3236 (86.4)	1372 (81.5)	618 (78.8)	
Bisexual	803 (12.9)	415 (11.1)	262 (15.6)	126 (16.1)	
Heterosexual	63 (1.0)	36 (1.0)	18 (1.1)	9 (1.1)	
Other	121 (1.9)	59 (1.6)	31 (1.8)	31 (4.0)	
*Missing* ^a^	7 (0.0)	3 (0.0)	2 (0.0)	2 (0.0)	
**Education**					< 0.001
Elementary or less	136 (2.2)	73 (1.9)	48 (2.8)	15 (1.9)	
Secondary	1365 (21.9)	684 (18.2)	448 (26.6)	233 (29.6)	
Terciary or higher	4692 (75.4)	2981 (79.5)	1180 (70.0)	531 (67.6)	
Preferred not to answer	27 (0.4)	11 (0.3)	9 (0.5)	7 (0.9)	
**Monthly family income^1^**					< 0.001
Low	1393 (22.4)	638 (17.0)	500 (29.7)	255 (32.4)	
Middle	2740 (44.1)	1621 (43.2)	752 (44.6)	367 (46.7)	
High	1872 (30.1)	1338 (35.7)	387 (23.0)	147 (18.7)	
Preferred not to answer	215 (3.5)	152 (4.1)	46 (2.7)	17 (2.2)	
**Country region**					< 0.001
North	100 (1.6)	46 (1.2)	47 (2.8)	7 (0.9)	
North East	717 (11.5)	275 (7.3)	306 (18.2)	136 (17.3)	
Midwest	392 (6.3)	201 (5.4)	143 (8.5)	48 (6.1)	
Southeast	4300 (69.1)	2702 (72.1)	1055 (62.6)	543 (69.1)	
South	711 (11.4)	525 (14.0)	134 (8.0)	52 (6.6)	
**Living in a state capital**					< 0.001
No	1657 (26.6)	1082 (28.9)	408 (24.2)	167 (21.2)	
Yes	4563 (73.4)	2667 (71.1)	1277 (75.8)	619 (78.8)	
**Illicit substance use**					0.013
No	4001 (64.3)	2368 (63.2)	1133 (67.2)	500 (63.6)	
Yes	2219 (35.7)	1381 (36.8)	552 (32.8)	286 (36.4)	
**Binge drinking**					<0.001
No	4351 (70.0)	2716 (72.4)	1147 (68.1)	488 (62.1)	
Yes	1869 (30.0)	1033 (27.6)	538 (31.9)	298 (37.9)	
**HIV status**					0.68
Negative	4221 (67.9)	2567 (68.5)	1123 (66.7)	531 (67.6)	
Positive	1590 (25.6)	934 (24.9)	450 (26.7)	206 (26.2)	
Unknown	407 (6.5)	247 (6.6)	111 (6.6)	49 (6.2)	
*Missing* ^*a*^	2 (0.0)	1 (0.0)	1 (0.0)	0 (0)	
**Internalized homonegativity score^2^ (mean; SD)**	10.3 (8.2)	10.1 (8.2)	10.9 (8.3)	9.6 (8.0)	< 0.001
**Discrimination score^3^ (mean; SD)**	3.5 (3.8)	3.1 (3.4)	3.7 (3.9)	5.2 (4.6)	< 0.001
**Depressive symptoms (PHQ-9)** ^4^					
Mean scores (SD)	8.8 (7.1)	8.4 (7.0)	9.3 (7.2)	9.6 (7.5)	<0.001
No symptoms	2030 (33.2)	1296 (35.1)	501 (30.4)	233 (30.3)	<0.001
Mild symptoms	1961 (32.1)	1203 (32.6)	518 (31.5)	240 (31.2)	
Moderate symptoms	882 (14.4)	512 (13.9)	249 (15.1)	121 (15.7)	
Moderate to severe symptoms	647 (10.6)	354 (9.6)	215 (13.1)	78 (10.1)	
Severe symptoms	587 (9.6)	326 (8.8)	163 (9.9)	98 (12.7)	

IQR: interquartile range, SD: standard deviation, PHQ-9: Patient Health Questionnaire with 9 items

^1^Monthly family income based on the minimum wage (R$1,212.00 in 2021, equivalent to US$242.00)

^2^Reactions to Homosexuality Scale

^3^Explicit Discrimination Scale

^4^No symptoms (0–4 points); mild symptoms (5–9 points); moderate symptoms (10–14 points); moderate to severe symptoms (15–19 points); severe symptoms (20–27 points)

^a^*Missing*: missing information provided for all variables that had missing data

Mean discrimination score among all participants was 3.5 (SD 3.8) with the highest mean among Black participants (5.2, SD 4.6) compared to *Pardo* (3.7, SD 3.9) and White (3.1, SD 3.4) participants (p < 0.0001). Mean internalized homonegativity score was 10.3 (SD 8.2) overall with the highest mean among *Pardo* participants (10.9, SD 8.3) compared to White (10.1, SD 8.2) and Black (9.6, SD 8.0) participants (p < 0.0001). Sexual orientation was frequently reported as a reason for discrimination across the three racial groups (∼63%) (Table 2). Most Black participants (77.9%) reported race as a reason for discrimination. Compared to White participants (24.8%), Black (52.8%) and *Pardo* (41.9%) participants more frequently identified their social class as a reason for discrimination. Housing location and religion were more frequently reported as reasons for discrimination by Black participants than by *Pardo* or White participants.

**Table 2:** Reported reasons for discrimination across the 8-items of the Explicit Discrimination Scale stratified by race.

	White (N,%)	Pardo (N,%)	Black (N,%)
**Total**	3749 (100.0)	1685 (100.0)	786 (100.0)
Race	97 (2.6)	548 (32.5)	612 (77.9)
Social class	928 (24.8)	706 (41.9)	415 (52.8)
Sexual orientation	2366 (63.1)	1043 (61.9)	498 (63.4)
Gender	540 (14.4)	268 (15.9)	131 (16.7)
Disease	87 (2.3)	44 (2.6)	12 (1.5)
Age	458 (12.2)	247 (14.7)	129 (16.4)
Housing location	251 (6.7)	216 (12.8)	130 (16.5)
Accent	498 (13.3)	300 (17.8)	114 (14.5)
Clothing	51 (1.4)	14 (0.8)	2 (0.3)
Overweight	550 (14.7)	244 (14.5)	103 (13.1)
Disability	49 (1.3)	30 (1.8)	14 (1.8)
Appearance	66 (1.8)	28 (1.7)	4 (0.5)
Political beliefs	433 (11.5)	189 (11.2)	99 (12.6)
Religion	224 (6.0)	136 (8.1)	85 (10.8)
Other	133 (3.5)	39 (2.3)	14 (1.8)

Mean PHQ-9 score was 8.8 (SD 7.1) with the highest mean among Black participants (9.6, SD 7.5), followed by Pardo (9.3, SD 7.2) then White (8.4, SD 7.0) participants (p < 0.0001). A higher proportion of White participants reported no symptoms of depression compared to *Pardo* and Black participants (35.1%, 30.4%, and 30.3%, respectively). Considering the 10 points cut-off, a higher proportion of Black and *Pardo* participants reached the cut-off compared to White participants (38.5%, 38.1%, and 32.3%, respectively). Overall, more than half of participants reported endorsing most of PHQ-9 items for several days, more than half the days, or almost every day, except for items 8 (moving or speaking so slowly that other people could have noticed or the opposite) and 9 (thoughts that you would be better off dead, or of hurting yourself) (Fig 2).

**Fig 2:**
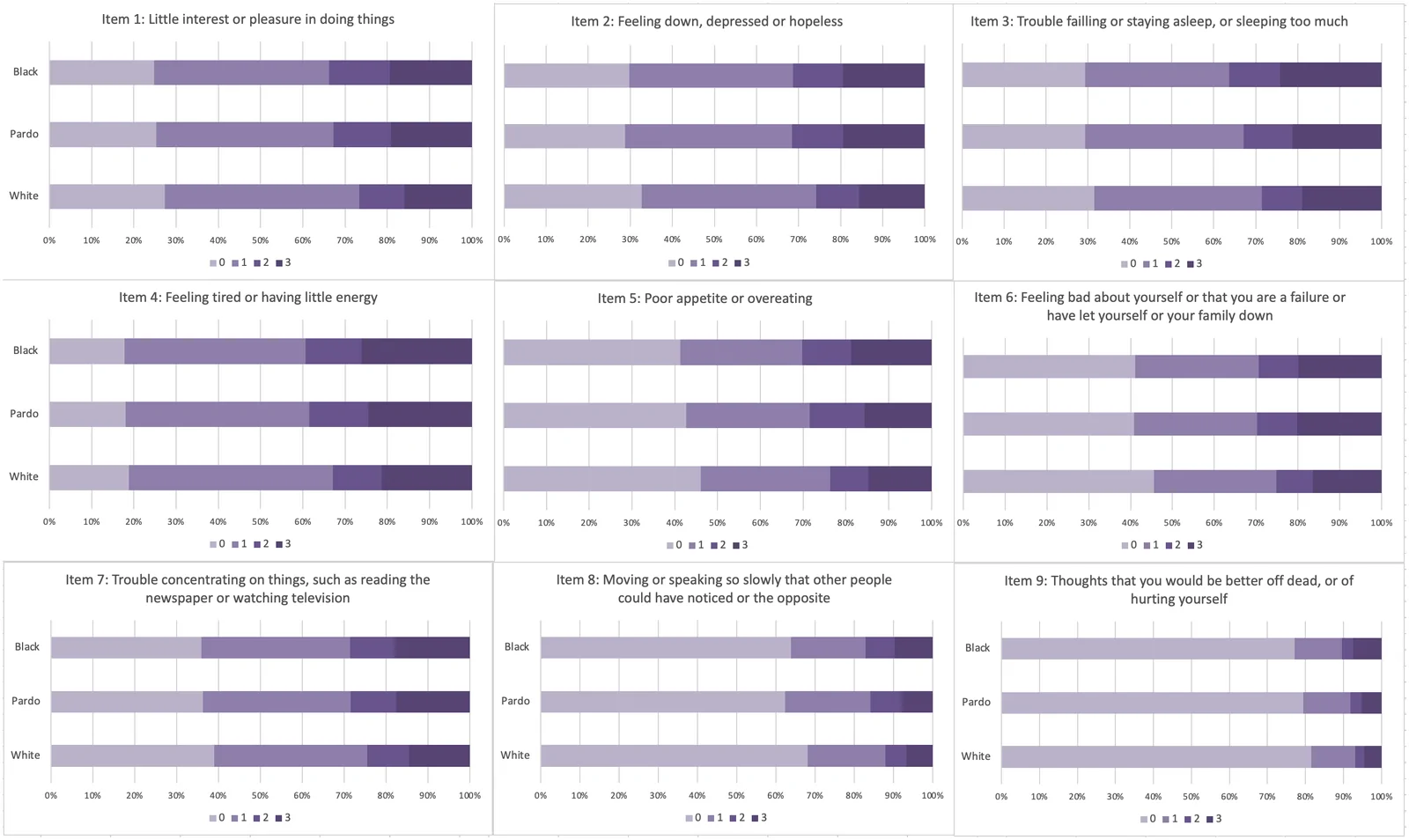
Percentage of persons reporting frequency of symptoms for each PHQ-9 item according to race, Brazil, November 2021 to January 2022. Legend: 0: not at all; 1: several days; 2: more than half the days; 3: almost every day.

When assessing the effect of the discrimination score, results showed that a one SD increase in the discrimination score was associated with higher scores of depression symptoms for all races (p < 0.0001) (Table 3). When evaluating the effect of internalized homonegativity, results showed that a one SD increase in the internalized homonegativity score was associated with higher scores of depression symptoms only among White (p = 0.045) and *Pardo* (p = 0.035) participants. The model combining discrimination and internalized homonegativity scores showed that both variables retained their original effect sizes, with the effect size for discrimination being substantially higher than internalized homonegativity. Neither effect changed by the presence of the other variable. Including other covariates in the models resulted in a slight decrease in the effect size for discrimination score and slight increase in the effect size for internalized homonegativity. The adjusted models also showed an association between younger age and higher scores of depression symptoms regardless of race. Lower income was associated with higher scores of depression symptoms for *Pardo* and White participants, but not for Black participants.

**Table 3:** Linear regression models’ results assessing the association between discrimination score (measured by Explicit Discrimination Scale), internalized homonegativity (measured by the Reactions to Homosexuality Scale), and other co-variables with depressive symptoms measured by the Patient Health Questionnaire (9 items), stratified by race.

	White		*Pardo*		Black	
	Estimate (SD)	p-value	Estimate (SD)	p-value	Estimate (SD)	p-value
***Univariable models of main exposures***						
**Discrimination score** ^**2**^	**0.40 (0.02)**	**<0.0001**	**0.36 (0.02)**	**<0.0001**	**0.32 (0.03)**	**<0.0001**
**Internalized homonegativity score** ^**3**^	**0.03 (0.02)**	**0.045**	**0.05 (0.02)**	**0.035**	-0.01 (0.04)	0.72
***Multivariable model (main exposures combined)***						
**Discrimination score**	**0.39 (0.02)**	**<0.0001**	**0.36 (0.02)**	**<0.0001**	**0.32 (0.03)**	**<0.0001**
**Internalized homonegativity score**	**0.04 (0.01)**	**0.0072**	**0.05 (0.02)**	**0.030**	-0.01 (0.04)	0.71
***Multivariable model (main exposures and co-variables combined)***						
**Discrimination score**	**0.36 (0.02)**	**<0.0001**	**0.34 (0.03)**	**<0.0001**	**0.30 (0.03)**	**<0.0001**
**Internalized homonegativity score**	**0.08 (0.02)**	**<0.0001**	**0.08 (0.02)**	**0.0011**	0.01 (0.04)	0.71
**Age (per 10-year increase)**	**-0.18 (0.01)**	**<0.0001**	**-0.21 (0.03)**	**<0.0001**	**-0.24 (0.04)**	**<0.0001**
**Gender**						
Cisgender man	Ref.		Ref.		Ref.	
Trans or non-binary person	1.19 (0.12)	0.10	0.04 (020)	0.84	0.37 (0.23)	0.10
**Sexual orientation**						
Gay	Ref.		Ref.		Ref.	
Other	0.01 (0.05)	0.91	0.09 (0.07)	0.76	-0.05 (0.10)	0.62
**Education**						
Elementary or less	-0.02 (0.12)	0.87	-0.01 (0.15)	0.92	0.02 (0.26)	0.94
Secondary	0.04 (0.04)	0.28	0.02 (0.06)	0.76	0.10 (0.08)	0.26
Terciary	Ref.		Ref.		Ref.	
**Monthly family income¹**						
Low	**0.19 (0.05)**	**<0.0001**	**0.29 (0.07)**	**<0.0001**	0.15 (0.11)	0.18
Middle	**0.11 (0.03)**	**<0.0001**	0.07 (0.06)	0.24	-0.01 (0.10)	0.90
High	Ref.		Ref.		Ref.	
**Region**						
North, Northeast or Midwest	-0.03 (0.04)	0.55	-0.03 (0.05)	0.52	0.06 (0.08)	0.45
Southeast or South	Ref.		Ref.		Ref.	
**Lives in a state capital**						
No	0.01 (0.03)	0.84	-0.03 (0.05)	0.56	0.02 (0.09)	0.77
Yes	Ref.		Ref.		Ref.	
**Illicit substance use**						
No	Ref.		Ref.		Ref.	
Yes	0.01 (0.03)	0.69	0.06 (0.05)	0.27	0.14 (0.08)	0.065
**Binge drinking**						
No	**Ref.**		Ref.		Ref.	
Yes	**0.08 (0.04)**	**0.026**	0.09 (0.05)	0.076	0.05 (0.08)	0.51
**HIV status**						
Negative	Ref.		Ref.		Ref.	
Positive	0.01 (0.04)	0.89	0.03 (0.05)	0.56	0.03 (0.08)	0.74
Unknown	-0.01 (0.06)	0.89	-0.03 (0.10)	0.74	-0.08 (0.15)	0.60

SD: standard deviation. **Bold**: p < 0.05; ¹ Monthly family income based on the minimum wage (MW) which was R$1,212.00 in 2021 (US$242.00); ² Reactions to Homosexuality Scale; ^³^ Explicit Discrimination Scale

## Discussion

In this study, we found that Black SGD participants from Brazil had a higher prevalence of depressive symptoms (also seen for *Pardo* SGD participants), higher discrimination scores, and lower internalized homonegativity scores compared to their White counterparts. We found that discrimination was associated with depressive symptoms among all races, corroborating findings from a study among 489 transgender women attending an HIV prevention and care service in Rio de Janeiro, Brazil [35]. Depressive symptoms were associated with experiences of discrimination and internalized homonegativity, as well as sociodemographic characteristics including age and income among White and *Pardo* participants.

Using a cut-off of 10 points in the PHQ-9 scale, we observed high rates of depressive symptoms in our sample (34.6% overall, 32.3% among White, 38.1% among Pardo, and 38.5% among Black participants), corroborating previous studies with SGD in Brazil [36,37]. According to the 2019 Brazilian National Health Survey (PNS 2019), which included self-reported data on the diagnosis of depression given by a mental health professional, an estimated 10.2% of the Brazilian population aged 18 years or older reported having received a clinical diagnosis of depression, which is much lower than the ~ 30% observed in our study [38]. Compared to the previous national survey, carried out in 2013 (PNS 2013), an increase in the prevalence of depression was observed, from 7.9% in 2013 to 10.8% in 2019 [39]. This increase was mainly observed among young adults who reported as unemployed [39]. Multiple reasons might explain the higher prevalence of depressive symptoms observed in our study including our focus of SGD persons, that our study was conducted at the end of 2021 and early 2022 (during COVID-19 pandemic), and the use of PHQ-9 to assess the presence of depressive symptoms instead of a diagnosis given by a mental health professional.

In our study, Black and *Pardo* SGD participants had more depressive symptoms than those who self-declared as White. Racism, everyday discrimination, reduced access to mental health care, and psychosocial stressors may explain these racial disparities [40]. As shown in our study, Black and *Pardo* SGD participants more frequently identified their social class as a reason for discrimination than White participants. Interestingly, the PNS 2019 observed higher rates of clinically diagnosed depression among White persons compared to Black and Pardo persons in Brazil [38]. Additionally, PNS 2019 respondents reported that mental health care was predominantly accessed through private healthcare services [38]. These results suggest that disparities in access to mental health services experienced by racially and socially marginalized groups which may be potentially leading to an underdiagnoses of depression among non-White persons [41].

Discrimination scores were considerably higher among Black SGD participants, who more frequently identified race as a reason for discrimination, highlighting that racial discrimination may be the most significant stress factor. The enslavement of Africans was not limited to the violent trafficking and exploitation of people, it unfolded in the construction of subjectivities of Black persons marked by the introjection of representations and beliefs of inferiority, impacting Black subjectivities [42,43]. Additionally, the construction of Black people’s identities may be shaped by the contempt and hatred historically projected by whiteness, which is often regarded as the standard of success, beauty, and humanity [42,43]. When internalized, this symbolic violence leads to a process of self-hatred, in which Black individuals begin to reject aspects of their own identity, including physical and cultural attributes associated with Blackness [42]. This process tends to result in the reproduction of discriminatory behaviors among peers [44], intense psychological suffering, unhealthy social relationships, distorted self-image, and depressive symptoms [40]. Although racism is one of the structuring aspects of Brazilian inequalities, the lack of institutionalization of racism – such as apartheid in South Africa – may mistakenly suggest that socioeconomic and not racial inequalities prevail. This results in silencing around racism and its effects, meaning that pain and violence are not expressed, spoken or recognized, making it difficult to care for and work through the traumatic experiences produced by racial discrimination [45]. Additionally, Black MSM, are often associated with stereotypes that portray them as having a hypersexualized, impulsive and violent body, characteristics also associated with heterosexual Black men [46].

Internalized homonegativity was associated with depression among White and Pardo, but not Black SGD participants. SGD persons may face specific challenges related to understanding their sexual orientation and/or gender identity and accepting themselves, and they may be exposed to discriminatory situations, hostile environments and rejection or lack of family support [18]. By not experiencing acceptance in their social circles, they may tend to internalize prejudice, resulting in feelings of shame, isolation and exclusion behaviors and suicidal thoughts [13]. The absence of the association between internalized homonegativity and depressive symptoms among Black SGD participants suggests that the predominant factors differ between the racial groups. That said, it is important to note that the effect size for the association between discrimination and depressive symptoms was much higher than that for the association between internalized homonegativity and depressive symptoms, suggesting that, at least in our sample, discrimination plays a much larger role regardless of race. In a cross-sectional online Brazilian study with 926 respondents enrolled from August-November 2020, internalized homonegativity was positively associated with depression among those reporting affective orientation towards same-gender [47]. A respondent-driven sampling study conducted in 12 Brazilian cities in 2016 observed that moderate or high/very high discrimination due to sexual orientation increased the odds of experiencing moderate/severe depressive symptoms [37]. In a longitudinal study from the United States that included young Black and Latino sexual minority men, both racial discrimination and internalized homonegativity were associated with depressive symptoms [48].

The association between younger age and depressive symptoms among SGD participants is consistent with previous studies that point to the greater vulnerability of young SGD persons to mental health issues [49–51]. In the general population, being a young adult can be considered a risk factor for depression, since the transition from adolescence to adulthood is marked by intense changes and can be understood as a crisis in search of a new identity [52]. A systematic review of 42 systematic reviews and meta-analyses reported on the prevalence, severity, and risk factors for mental disorders among SGD persons aged 25 years or lower. Its results showed that SGD youth experienced a heightened risk of depression and a greater severity of symptoms compared to their heterosexual/cisgender peers [53]. In addition, when evaluating suicide attempt, a frequent depression downstream event, another systematic review and meta-analysis of studies published between January 1990 and June 2016 suggested a higher risk of suicide attempts among gay and bisexual persons aged 12–26 years compared to their heterosexual counterparts [54].

Our results also showed an association between low income and depressive symptoms among *Pardo* and White SGD participants. Access to basic resources and opportunities have a direct impact on the mental health status of the SGD population [55]. SGD persons are often affected by socioeconomic inequalities, lack of access to the labor market, and discrimination in the workplace [56]. The economic inequalities that affect SGD populations are multifaceted and based on gender and sexual discrimination. This may involve the loss of family networks with early leaving or expulsion from home, low learning outcomes and school dropouts, difficulties in accessing and remaining in vocational or undergraduate courses, migration to self-employed or informal jobs, and high rates, especially among trans people, of exploitative sex work [57]. Low income is one of the main barriers to accessing mental health care and, with the scarcity of these services in the public health system, SGD persons with financial difficulties often do not access mental health care [58].

This study has strengths and limitations to be considered when interpreting the results. PHQ-9 is a validated, recognized, and widely used tool to assess depressive symptoms in the past two weeks, nevertheless, it is not diagnostic of a depressive disorder, which may only be adequately assessed in consultations with a mental health professional. Though discrimination and internalized homonegativity are complex, latent constructs, we used instruments that have been validated in a Brazilian context. Due to low sample size, we were unable to include Indigenous and Asian participants in this analysis. Additionally, also due to the small samples, we aggregated all SGD persons in only one group and did not consider the specificities of each gender identity and sexual orientation. This analysis accessed individuals who were available to complete the survey, meaning they had internet access and sufficient time. This may have contributed to the greater participation of individuals with higher socioeconomic status. As a cross-sectional study, it is not possible infer temporality nor causality. We recruited a convenience sample of SGD persons through apps and social media, precluding extrapolation of our findings to the overall SGD population in Brazil. Lastly, sample mean age was 36 years, which is comparable to other studies using online samples [29,59]. Future studies should use other strategies to reach and retain younger SGD persons.

## Conclusions

We found a high burden of depressive symptoms among a sample of SGD persons from Brazil. The burden of depressive symptoms varied by race, being higher among those of Black or *Pardo* race. Reporting more experiences of discrimination was positively associated with higher PHQ-9 scores regardless of race. As we advocate for equity, it is crucial to acknowledge and address the unique challenges faced by racial minorities and SGD persons. Reducing vulnerability to depression among SGD persons requires psychosocial approaches that consider the intersecting impacts of racism, homophobia, and transphobia on mental health. Structural interventions must be combined to inclusive, affirming, and equitable mental health services that foster emotional resilience and adaptive coping. Such services are essential to mitigate the cumulative psychological burden experienced by SGD persons, who have historically encountered stigma-related barriers to health information, services, and prevention.

## Supporting information

S1 Data Dataset. Id: participant number; phq9_1 to phq9_10: scores for each item of the Patient Health Questionnaire-9 (PHQ-9); phq_score: total score for PHQ-9.
(XLSX)
