# Supplementary material for: High burden of depressive symptoms among sexual and gender diverse persons in Brazil: The role of discrimination, internalized homonegativity, and race
Source: PLOS Glob Public Health. 2026 Sep 28;6(9):e0007276. doi: 10.1371/journal.pgph.0007276 (PMC13618917; doi:10.1371/journal.pgph.0007276)

PLOS Global Public Health

High burden of depressive symptoms among sexual and gender diverse individuals of Black race in Brazil: the role of discrimination and internalized homophobia

--Manuscript Draft--

Manuscript Number:	PGPH-D-26-00431
Article Type:	Research Article
Full Title:	High burden of depressive symptoms among sexual and gender diverse individuals of Black race in Brazil: the role of discrimination and internalized homophobia
Short Title:	Depressive symptoms and discrimination on LGBTQIAPN+ populations
Corresponding Author:	Thiago Silva Torres, Ph.D.<br>FIOCRUZ: Fundacao Oswaldo Cruz<br>Rio de Janeiro, BRAZIL
Order of Authors:	Caroline Guimaraes
	Paula Mendes Luz
	Carolina Coutinho
	Emilia M. Jalil
	Cristina M. Jalil
	Beatriz Grinsztejn
	Valdilea G. Veloso
	Lucilene Araujo Freitas
	Thiago Silva Torres, Ph.D.
Keywords:	Depression; racism; LGBTQIAPN+; MSM; transgender; Latin America
Abstract:	Sexual and gender diverse (SGD) populations have historically faced restricted rights and systematic discrimination for deviating from the established hetero-cis-normative norms. This long-standing marginalization has a profound negative impact on the health outcomes, including a heightened risk of developing mental health issues, such as depression. Understanding the health-disease process among SGD populations requires recognizing that discriminatory experiences, such as homophobia and transphobia, are not unidimensional, but operate synergistically with layered forms of discrimination, such as racism. We assessed depressive symptoms using the Patient Health Questionnaire-9 (PHQ-9) among SGD individuals in Brazil using race as a stratification variable. We also evaluated the role of discrimination and internalized homophobia on depressive symptoms, while adjusting for covariates. This was a cross-sectional online survey recruiting SGD individuals aged 18 years or older, living in Brazil, through dating apps and social media. Overall, 6220 participants were included, 3749 (60.3%) White, 1685 (27.1%) Pardo and 786 (12.6%) Black. Mean scores of depressive symptoms was 8.8 (SD7.1), higher among Black (9.6; SD 7.5) and Pardo (9.3; SD 7.2) than White (8.4; SD7.0) (p<0.0001). Considering the PHQ-9 cut-off of ≥ 10 points, more Black and Pardo participants present depressive symptoms than White (38.5%, 38.1%; 32.3%, respectively). In multivariable linear regression models, higher scores of discrimination and younger age were associated with higher scores of depressive symptoms for all races. Internalized homophobia and lower income were associated with higher depressive symptoms scores for Pardo and White, but not for Black participants. To effectively address vulnerability to depression among SGD populations, it is essential to establish approaches that consider the intersections between different forms of oppression. This includes policies that tackle racism, homophobia, and transphobia, as well as investment in accessible mental health services.
Additional Information:	
**Question**	**Response**

Powered by Editorial Manager® and ProduXion Manager® from Aries Systems Corporation

**Financial Disclosure**<br><br>Enter a financial disclosure statement that describes the sources of funding for the work included in this submission and the role the funder(s) played. This includes grants and any commercial funding of the work or authors.<br><br>This statement will be typeset if the manuscript is accepted for publication.<br><br>*Please review the submission guidelines and the instructions link below for detailed requirements and guidance.*	T.S.T. was supported by Conselho Nacional de Desenvolvimento Científico e Tecnológico (CNPq; #304417/2025-4 and #405558/2025-2. P.M.L. was supported by CNPq (#30700/2025-2) and Fundação Carlos Chagas Filho de Amparo à Pesquisa do Estado do Rio de Janeiro (FAPERJ; #E-26/204.206/2024). B.G. was supported by CNPq (#313265/2023-2) and FAPERJ (#E.26/200.946/2022). The authors declare no conflicts of interest. The funding body had no role in study design, data collection, data analysis, or data interpretation.
**Competing Interests**<br><br>On behalf of all authors, disclose any competing interests that could be perceived to bias this work.<br><br>This statement will be typeset if the manuscript is accepted for publication.<br><br>*Please review the instructions link below and PLOS Global Public Health's competing interests policy to determine what information must be disclosed at submission.*	The authors declare no competing interests.
**Data Availability**<br><br>Before publication, Authors are required to make fully available and without restriction all data underlying their findings. Please see our PLOS Data Policy page for detailed information on this policy.<br><br>A **Data Availability Statement**, detailing where the data can be accessed, is required at first submission. Insert your	A complete de-identified dataset sufficient to reproduce this analysis will be made available upon request to the corresponding author, following approval of a concept sheet summarizing the analyses to be done.

Data Availability Statement in the box below.<br><br>Please see the data reporting section of our submission guidelines for instructions on what you need to include in your Data Availability Statement.<br><br>This statement will be typeset if the manuscript is accepted for publication.<br><br>*PLOS allows rare exemptions to address legal and ethical concerns. If you have legal or ethical restrictions, please use the box below to detail these in full sentences for the Journal team to consider.*	

Manuscript
Click here to access/download;Manuscript;guimaraes_01feb26.docx

Title Page

High burden of depressive symptoms among sexual and gender diverse individuals of Black race in Brazil: the role of discrimination and internalized homophobia (160/200 characters)

Depressive symptoms and discrimination on LGBTQIAPN+ populations (64/70 characters)

Caroline Guimarães,[1] Paula Mendes Luz,[1] Carolina Coutinho,[1] Emilia M. Jalil,[1] Cristina M. Jalil,[1] Beatriz Grinsztejn,[1] Valdilea Gonçalves Veloso,[1] Lucilene Araújo Freitas,[1]Thiago Silva Torres[1*]

[1] Evandro Chagas National Institute of Infectious Diseases, Oswaldo Cruz Foundation

* Corresponding author

Av Brasil 4365 Manguinhos, 21040-360

Rio de Janeiro, RJ, Brazil

thiago.torres@ini.fiocruz.br

ORCID: 0000-0002-2557-601X

Authors' contribution

CG, PML, LAF and TST conceived the study. CG, PML and TST conceived and supervised this analysis. LAF and CG had access to, and verified, the data. TST did the statistical analyses. CC drafted the manuscript. CC, EMJ, CMJ, BG and VGV helped with data acquisition and interpretation

of the findings. All authors were involved in revising the manuscript for important intellectual content. All authors read and approved the final manuscript.

Abstract (284/300 words)

Sexual and gender diverse (SGD) populations have historically faced restricted rights and systematic discrimination for deviating from the established hetero-cis-normative norms. This long-standing marginalization has a profound negative impact on the health outcomes, including a heightened risk of developing mental health issues, such as depression. Understanding the health-disease process among SGD populations requires recognizing that discriminatory experiences, such as homophobia and transphobia, are not unidimensional, but operate synergistically with layered forms of discrimination, such as racism. We assessed depressive symptoms using the Patient Health Questionnaire-9 (PHQ-9) among SGD individuals in Brazil using race as a stratification variable. We also evaluated the role of discrimination and internalized homophobia on depressive symptoms, while adjusting for covariates. This was a cross-sectional online survey recruiting SGD individuals aged 18 years or older, living in Brazil, through dating apps and social media. Overall, 6220 participants were included, 3749 (60.3%) White, 1685 (27.1%) *Pardo* and 786 (12.6%) Black. Mean scores of depressive symptoms was 8.8 (SD7.1), higher among Black (9.6; SD 7.5) and *Pardo* (9.3; SD 7.2) than White (8.4; SD7.0) ($p<0.0001$). Considering the PHQ-9 cut-off of ≥ 10 points, more Black and *Pardo* participants present depressive symptoms than White (38.5%, 38.1%; 32.3%, respectively). In multivariable linear regression models, higher scores of discrimination and younger age were associated with higher scores of depressive symptoms for all races. Internalized homophobia and lower income were associated with higher depressive symptoms scores for *Pardo* and White, but not for Black participants. To effectively address vulnerability to depression among SGD populations, it is essential to establish approaches that consider the intersections between different forms of oppression. This includes policies that tackle racism, homophobia, and transphobia, as well as investment in accessible mental health services.

Keywords: Depression, Racism, LGBTQIAPN+, MSM, transgender, Latin America

Introduction

Depression is a common mental disorder, affecting an estimated 5.7% of adults globally [1]. Despite the availability of effective and recognized treatments, more than 75% of people with depression do not have access to care in low- and middle-income countries, mainly due to insufficient investments in mental health care and the stigma surrounding mental disorders [2]. As a multifactorial phenomenon, depression is not understood as an essentially individual condition, but rather as the result of the interaction between genetic, psychological, and environmental factors, which can be influenced by stressful events such as loss of loved ones, discrimination, and violence [3].

Stigma, prejudice, and discrimination are interconnected social phenomena that produce suffering and act as social determinants of health [4]. Stigma is the association of a person with a stereotype or an attribute that society deems undesirable, reducing them to a single, discredited trait [5,6]. Prejudice is defined as a hostile attitude towards a person simply because they belong to a certain group resulting from the assumption that they possess the negative characteristics that are attributed to the group [6]. Both stigma and prejudice involve categorization, labelling, stereotyping and social rejection, resulting in discrimination and exclusion [7].

Associated with stigma and prejudice, discrimination refers to the practice or behavior of unfair or unequal treatment based on personal characteristics or perceived identities, such as race, ethnicity, gender, sexual orientation, socioeconomic status, among others [8]. This differential treatment can manifest itself in a variety of ways, including marginalization, exclusion, harassment and violence. Discrimination can occur at the individual level, being reproduced in interpersonal interactions, and at structural levels, in policies, laws and institutions that perpetuate inequalities and injustices that affect certain groups [4]. Considering the impossibility of analyzing in isolation the factors that affect individuals' lives, intersectionality provides visibility to the inequalities that impact the

existence of certain marginalized groups who are systematically exposed to discriminatory situations, and it allows for the development of responses that address their specific needs [9].

Historically, sexual and gender diverse (SGD) populations have had their rights restricted and have been victims of discrimination for diverging from the established heterosexual norm. This constant exposure to discrimination may have a negative impact on the physical and mental health of these populations [10]. To understand the health–illness process among SGD populations, it is essential to recognize that different forms of discrimination affect this group, such as lesbophobia, homophobia, biphobia, and transphobia. These forms of discrimination do not occur in isolation but are reinforced and intersect with other forms of prejudice, such as racism, sexism, and misogyny [11]. Among Black SGD individuals, various intersections can be observed, such as an increased risk of violence due to skin color, gender identity, and sexual orientation, which directly impacts their psycho-emotional well-being [12]. In Brazil, systemic racism has persisted for centuries, resulting in social vulnerability and worse health outcomes for Black, *Pardo*, and Indigenous populations [13].

Discrimination increases the risk of developing psychiatric disorders, including major depressive disorders, which is defined by the "presence of a sad, empty or irritable mood, accompanied by somatic and cognitive changes that significantly affect the individual's ability to function" [3]. Both discrimination and internalized homophobia may constitute significant stressors in the lives of SGD individuals, potentially exerting a range of adverse effects on their mental health. Frequent exposure to prejudice leads to the internalization of negative messages about one's own sexual and gender identity, which can result in low self-esteem and feelings of self-hatred, causing these individuals to withdraw from their communities and avoid social situations that could expose them to the risk of discrimination [14]. This constant worry about the possibility of being the target of prejudice or violence can contribute to feelings of hopelessness, sadness and isolation, increasing the risk of depression or other mental health disorders [15].

This study assessed the depressive symptoms among SGD individuals in Brazil using race as stratification variable to understand the unique experiences of each racial group. Additionally, we aim to investigate the association of discrimination, internalized homophobia and other co-variables with depressive symptoms, stratified by race.

Materials and methods

Study design and questionnaire

This was a cross-sectional online survey enrolling a convenience sample recruited through dating apps (Grindr, Hornet and Scruff) and social media (Facebook and Instagram) from 07/11/2021 to 29/01/2022. Invitations to voluntarily participate in the survey were sent via direct messages on dating apps and boosted posts on social media. Details of study design and results from the primary analysis are described elsewhere [8]. Inclusion criteria were: a) self-reporting as a person of a SGD identity, b) age 18 years and older, and c) living in Brazil. Exclusion criteria were: a) failure to provide informed consent; b) identifying as a cisgender woman; c) identifying as cisgender men but reporting never having had sexual relations with other men; d) answering any of the attention questions incorrectly; and e) not reaching the end of the questionnaire. For this analysis, we additionally excluded those who: a) identified as Asian (n=103) and Indigenous (n=73) persons due to the low numbers, b) had missing information on race (n=129), and c) did not complete the Patient Health Questionnaire-9 (PHQ-9) (Figure 1). The study questionnaire included questions about sociodemographic characteristics, substance use, binge drinking, HIV status, discrimination, internalized homophobia, and depressive symptoms. The questionnaire was in Portuguese, and a non-response option was available for all items.

The study was approved by the Institutional Review Board at Instituto Nacional de Infectologia Evandro Chagas, Fundação Oswaldo Cruz (INI-Fiocruz; #CAAE 01777918.0.0000.5262). All study participants provided electronic informed consent before survey initiation. No compensation was provided.

Outcome

The PHQ-9, validated in Brazilian Portuguese [16], is a brief instrument widely used to screen individuals with severe depressive symptoms [17]. The instrument contains nine questions evaluating each symptom of a major depressive episode, presented in the Diagnostic and Statistical Manual of Mental Disorders (DSM-IV), and a tenth question that assesses the impact of these symptoms on daily functioning. The frequency of each symptom in the last two weeks was assessed on a *Likert* scale of 0 to 3 points corresponding to the answers "not at all" (0), "several days" (1), "more than half the days" (2) and "almost every day" (3). PHQ-9 scores are further classified into no symptoms (0-4), mild symptoms (5-9), moderate symptoms (10-14), moderate to severe symptoms (15-19), and severe symptoms (20-27). Nevertheless, high scores are not diagnostic; a definitive diagnosis can only be established through consultations with mental health professionals.

Main exposures

<u>Discrimination</u>

The Explicit Discrimination Scale (EDS) instrument, first developed with 18-items, assesses the occurrence of explicit discrimination in day-to-day situations that are applicable to the Brazilian context [18]. This instrument was the first to assess explicit discrimination outside the context of

high-income countries. To evaluate discrimination, it is essential to consider cultural, historical, and social contexts of a given society. In Brazil, social and racial conflicts often occur in a more subtle manner due to the myth of racial democracy, which has historically hidden inequalities [19]. The EDS is suitable for our analysis for three reasons: it addresses a wide range of situations that tend to be discriminatory; it allows participants to associate their experiences of discrimination with one or more factors simultaneously; and it considers that discrimination and the perception of a discriminatory event are distinct yet interrelated phenomena [20].

We used the abbreviated 8-item Explicit Discrimination Scale (EDS) which assesses perceived discrimination in 8 different circumstances. It has a robust capacity to address intersections between the different axes of inequality that interact and are experienced simultaneously [21]. Prior work has shown that the abbreviated EDS version was suitable for analysis of comparable experiences of discrimination among groups defined by sex, race, and education [20].

The abbreviated EDS contains the following items related to differential treatment: (1) treated disrespectfully in public places; (2) treated as unintelligent at school or college; (3) treated as unintelligent in the workplace; (4) unfair evaluation in workplace; (5) called names the person doesn't like; (6) excluded or left aside by friends at school; (7) excluded or left aside by coworkers; and (8) excluded or left aside by people in the neighborhood. Response options were (coding): never (0), occasionally (1), often (2), and always (3). Participant who reported a specific experience of differential treatment were asked whether they perceived it as discrimination (yes or no). If yes, responses to the first question, across the 8-items, were summed up yielding the participant's discrimination score, which ranged from 0 to 24. Higher scores indicated more discriminatory experiences. Participants also answered about the main reason for each discriminatory experience reported, with 12 pre-defined response options (race, social class, sexual orientation, gender, physical disability, age, residence location, accent, clothing, overweight, disease, appearance,

political orientation, religion, and other - which the participant needed to specify. Finally, individuals could indicate other reason(s) for the discriminatory experience.

Internalized homophobia

Internalized homophobia is a term used to describe negative feelings that a person may have towards themselves because of their own sexual orientation. This phenomenon occurs when individuals introject prejudices, stigmas, and negative social norms about their way of experiencing sexuality [22].

The Internalized Homophobia Scale (IHS) assesses three main dimensions: a) social comfort with gays, with two items measuring well-being in social situations with gays and places such as gay bars; b) public identification as gay and personal comfort with a gay identity, with two items measuring comfort with talking about homosexuality in public or being seen with an openly gay individual; and c) personal comfort with gay identity, with three items assessing comfort with one's own sexuality and the belief that homosexuality is as natural as heterosexuality [23]. The score is calculated by summing all items (ranging from 0 to 42). Higher scores indicate greater internalized homophobia. This instrument was validated in a sample of gay, bisexual and other men who have sex with men [24] and used in other studies with SGD populations from Brazil [25–27].

Co-variables

Age was described in median and interquartile range (IQR) and stratified as 18-24, 25-35, >35 years, following previous studies [8,26]. Race was categorized according to the definition of the Brazilian Institute of Geography and Statistics (IBGE): a) Asian, for people who identify themselves as Asian

or of Asian descent, such as Japanese, Chinese, Koreans and others; b) Black, for people who identify themselves as Black or of African descent; c) Indigenous, those who identify themselves as Indigenous, belonging to one or more of the various indigenous ethnic groups in Brazil; d) *Pardo*, people who declare themselves as mixed race or multiracial, in a combination of Black, Indigenous or White ancestry; and e) White, people who identify themselves as white or of European ancestry. *Pardo* is a specific race/color category used in Brazil that is characterized by a skin tone intermediate between black and white and by the consequent greater degree of mobility between these two chromatic extremes [28]. Gender was categorized in cisgender man, transgender man, transgender woman, *travesti* (in Latin America refers to a person who was assigned male at birth but identifies and expresses a feminine gender identity) and non-binary person. Sexual orientation refers to the internal experience related to affective-sexual attraction to another person [29]. This variable was collected as: a) asexual (absence of affective-sexual attraction to people of any gender); b) bisexual (affective-sexual attraction to people of any gender); c) gay or homosexual (affective-sexual attraction to people of the same gender as the one with which one identifies); d) heterosexual (affective-sexual attraction to people of a different gender to the one with which they identify); e) pansexual (affective-sexual attraction to people regardless of gender), and f) other. Education was categorized into elementary or less, secondary and tertiary or higher. Monthly family income was inquired in relation to the monthly minimum wage (MW), which was R$ 1,212 by the time of the study in Brazil and is equivalent to US$ 212. This variable was categorized into low (up to 2 MW), middle (more than 2 to 6 MW), and high (more than 6 MW). The Brazilian state of residence was recorded and categorized according to the country's administrative regions: North (seven states), Northeast (nine states), Midwest (three states and the Federal District), Southeast (four states) and South (three states). Definition of binge drinking was consuming five or more standard drinks (1 can of beer [340 mL] or 1 glass of wine [140 mL] or 1 shot of distilled alcohol [40 mL of ex. cachaça, vodka, whisky]) on a single occasion in the past year [30]. Substance use included any illicit

substance used in the past 6 months, such as marijuana, hash, cocaine, crack, amphetamines, lysergic acid (LSD) and ketamine ("special K"). Participants answered if they ever tested for HIV and, if yes, what was the result of their last test (negative, positive, or unknown).

Statistical analysis

First, we described the sample overall and according to self-identified race (Black, *Pardo* or White). We compared sociodemographic characteristics, binge drinking, substance use, HIV status, discrimination, internalized homophobia, and symptoms of depression (PHQ-9 mean scores and standard deviations [SD] and categories) according to participants' race using the Chi-square test for categorical variables and ANOVA and Kruskal-Wallis tests for continuous variables. Then, we described the proportion of individuals reporting "not at all" (0), "several days" (1), "more than half the days" (2) and "almost every day" per item of the PHQ-9 scale, according to race. We further describe the reported reasons for discrimination (either main or additional) across the 8 items of EDS stratified by race.

We adopt linear regression models stratified by race to assess if the exposures (discrimination and internalized homonegative) and other covariables (sociodemographic, binge drinking, substance use, and HIV status) were associated with the outcome. Models were stratified by race due to differential experiences of discrimination observed in prior work by our group, which found that Black SGD individuals experienced more discrimination compared to Pardo and White, with a unique contribution of racism to those disparities [8]. Our modeling approach included multiple sequential models, adding variables in a specific sequence to see how the effect size of your main exposures changes after including additional variables or confounders. We started investigating the association of discrimination with depression, followed by the association of internalized homophobia with

depression. Then, we assessed the association of discrimination and internalized homophobia with depression. Lastly, we added other covariates to this model. We evaluated the outcome in the model as a continuous variable, with higher scores of PHQ-9 indicating higher symptoms of depression. The PHQ-9 cut-off score of 10 or more has been used to estimate prevalence of depressive symptoms and to make treatment decisions individually. Nevertheless, evidence suggests that this cut-off substantially overestimates the prevalence and should be used with caution [31]. Due to its high sensitivity, the use of PHQ-9 score as a continuous variable proved to be more suitable than with the cut-off for screening major depressive symptoms in a Brazilian study [32]. Scale scores (outcome and factors) were standardized to enable comparisons of the model effect.

Results

Overall, 6220 participants were included in this analysis, 3749 (60.3%) self-identified as White, 1685 (27.1%) as *Pardo*, and 786 (12.6%) as Black (Table 1). Most were recruited on dating apps Grindr, Hornet and Scruff (90.6%). Median age was 36 years (interquartile range [IQR]: 29-44); most self-identified as cisgender men who have sex with men (MSM) (97.8%), as gay (84.1%), completed tertiary education (75.4%), had low or middle income (66.5%), were from the Southeast Brazil (69.1%), lived in a state capital (73.4%), and reported HIV negative status (67.9%). Substance use and binge drinking were reported by 35.7% and 30.0%, respectively. Compared to White individuals, a higher proportion of Black participants were younger, had a sexual orientation other than gay, lower education and income, lived in the Northeast region and in a state capital, and reported binge drinking (all $p < 0.001$).

Mean discrimination score among all participants was 3.5 (SD 3.8) with the highest mean among Black participants (5.2, SD 4.6) compared to those *Pardo* (3.7, SD 3.9) and White (3.1, SD 3.4)

(p<0.0001). Mean internalized homophobia score overall was 10.3 (SD 8.2) with the highest mean among *Pardo* participants (10.9, SD 8.3) compared to those White (10.1, SD 8.2) and Black (9.6, SD 8.0) (p<0.0001). Sexual orientation was frequently reported as a reason for discrimination across the three racial groups (~63%) (Table 2). Most Black participants (77.9%) reported race as a reason for discrimination. Compared to White participants (24.8%), more Black (52.8%) and *Pardo* (41.9%) participants identified their social class as a reason for discrimination. Housing location and religion were more frequently reported as reasons for discrimination by Black participants than by those self-declared as *Pardo* and White.

Mean symptoms of depression score was 8.8 (SD 7.1) with the highest estimate among Black participants (9.6, SD 7.5), followed by those Pardo (9.3, SD 7.2) and White (8.4, SD 7.0) (p<0.0001). More White participants reported no symptoms of depression compared to those Pardo and Black (35.1%, 30.4%, and 30.3%, respectively). Considering the 10 points cut-off, more Black and *Pardo* participants reported symptoms of depression compared to White participants (38.5%, 38.1%, and 32.3%, respectively). Overall, more than half of participants reported experiencing most of PHQ-9 symptoms for several days, more than half the days, or almost every day, except for items 8 (moving or speaking so slowly that other people could have noticed or the opposite) and 9 (thoughts that you would be better off dead, or of hurting yourself) (Figure 2). Compared to White participants, a higher proportion of Black and *Pardo* participants reported experiencing all items of the scale for at least several days within the past two weeks.

When assessing the effect of the discrimination score, the model showed that a one SD increase was associated with higher scores of depression symptoms for all races (p<0.0001) (Table 3). When evaluating the effect of internalized homophobia, the model showed that a one SD increase was associated with higher scores of depression symptoms only for White (p=0.045) and *Pardo* (p=0.035) participants. The model combining discrimination and internalized homophobia scores

showed that both variables retained their original effect sizes, with the effect size for discrimination being substantially higher. Neither effect changed by the presence of the other variable. Including other covariates in the model resulted in a slight decrease in the effect size for discrimination and slight increase in the effect size for internalized homophobia. The multivariable adjusted model also an association between younger age and higher scores of depression symptoms regardless of race. Lower income was associated with higher scores of depression symptoms for *Pardo* and White participants, but not for those self-declared as Black.

Discussion

In this study, Black SGD individuals from Brazil had a higher prevalence of depressive symptoms (also seen for *Pardo* SGD individuals), higher discrimination scores, and lower internalized homophobia scores compared to their White counterparts. Depressive symptoms were associated with experiences of discrimination and internalized homophobia, as well as sociodemographic aspects, such as age and income (for White and *Pardo* participants). Although it is a recognized social determinant of health in Brazil, this is one of the few studies to examine the intersection between race and mental health in SGD individuals.

We observed high rates of depression symptoms in our sample (34.6% overall, 32.3% among White, 38.1% among Pardo, and 38.5% among Black participants), corroborating previous studies with SGD in Brazil [33,34]. According to the 2019 Brazilian National Health Survey (PNS 2019), which included self-reported data on depression, an estimated 10.2% of the Brazilian population aged 18 years or older received a clinical diagnosis of depression from a mental health professional, much lower than observed in our study [35]. Compared to the previous survey, carried out in 2013, an

increase in the prevalence of depression was observed, from 7.9% in 2013 to 10.8% in 2019 [36]. This increase was mainly observed among young adults unemployed [36].

In our study, Black and *Pardo* SGD individuals had more depressive symptoms than those self-declared as White. Racism, everyday discrimination, reduced access to mental health care, and psychosocial stressors way explain these racial disparities [37]. These disparities are closely related to social disparities. As shown in our study, Black and *Pardo* SGD individuals more frequently identified their social class as a reason for discrimination than White individuals. Interestingly, the PNS 2019 observed higher rates of clinically diagnosed depression among White persons compared to Black and Pardo persons in Brazil [35]. Additionally, PNS 2019 respondents reported that mental health care was predominantly accessed through private healthcare services [35]. These results reinforce the ongoing disparities in access to mental health services experienced by racially marginalized groups and potentially influencing the underdiagnoses of depression [38].

Depressive symptoms result from a complex interaction between social, psychological, and biological factors. Persons who experience adverse experiences are more vulnerable to developing these symptoms [1]. In Brazil, as an effect of racism, a large part of the Black population lives in constant suffering due to precarious living conditions and the lack of prospects, which produces permanent anguish and may consequently lead to mental disorders including depression [39]. A systematic review, found a higher prevalence of depressive symptoms among Black women compared to White women, and among nonwhite older adults compared to their White counterparts in Brazil [40].

We found that discrimination was associated with depressive symptoms among all races. Association of discrimination and depressive symptoms was also found in a study among 489 transgender women attending an HIV prevention and care service in Rio de Janeiro, Brazil [41]. Since the 1990s, a series of studies have provided evidence for the development of a theoretical model for the understanding

of mental health conditions as experienced by people who suffer constant oppression [22]. The "minority stress theory", developed by the epidemiologist Ilan H. Meyer, was initially conceptualized for situations experienced by gay men in the U.S. Over time, however, the term has evolved to more broadly include various minority groups, such as other SGD persons, people with disabilities, Indigenous people and Black people. The theory represented an important advancement in understanding the mental health aspects of stigmatized groups and suggests that the more minority characteristics a person presents, the greater the damage to their mental health, implying an intersectional aspect. Additionally, the constant exposure to situations of prejudice and discrimination increases the risk of developing psychiatric issues in the groups affected. These include depression, which causes physical, psychological, emotional and social damage to the lives of affected individuals [42].

Discrimination scores were considerably higher among Black SGD individuals, who consistently identified race as a reason for discrimination, highlighting that racial discrimination may be the most significant stress factor. In this sense, the enslavement of Africans was not limited to the violent trafficking and exploitation of people, it unfolded in the construction of subjectivities of Black persons marked by the introjection of representations and beliefs of inferiority. Racism can also cause harm to Black subjectivities. The construction of Black people's identities may be shaped by the contempt and hatred historically projected by whiteness, which is often regarded as the standard of success, beauty, and humanity [43,44]. When internalized, this symbolic violence leads to a process of self-hatred, in which Black individuals begin to reject aspects of their own identity, including physical and cultural attributes associated with Blackness [43]. This process tends to result in the reproduction of discriminatory behaviors among peers [45], intense psychological suffering, unhealthy social relationships, distorted self-image, and consequent depressive symptoms [37]. Although racism is one of the structuring aspects of Brazilian inequalities, the lack of institutionalization of racism – such as apartheid in South Africa – may mistakenly suggest that

socioeconomic and not racial inequalities prevail. This results in silencing around racism and its effects, meaning that pain and violence are not expressed, spoken or recognized, making it difficult to care for and work through the traumatic experiences produced by racial discrimination [46]. Additionally, Black MSM, are often associated with stereotypes that portray them as having a hypersexualized, impulsive and violent body, characteristics also associated with heterosexual black men [47].

Internalized homophobia was associated with depression among White and Pardo, but not Black SGD individuals. SGD individuals may face specific challenges related to understanding their sexual orientation and/or gender identity and accepting themselves, as they may be exposed to discriminatory situations, hostile environments and rejection or lack of family support [15]. By not experiencing acceptance in their social circles, these young people may tend to internalize prejudice, resulting in feelings of shame, isolation and exclusion behaviors and suicidal thoughts [12]. The absence of the association of internalized homophobia and depression among Black MSG suggests that the predominant factors differ between the racial groups. The effect size for the association between discrimination and depression was much higher than that for the association between internalized homophobia and depression, suggesting that, at least in our sample, discrimination plays a large role regardless of race. In a cross-sectional online Brazilian study with 926 respondents enrolled from August-November 2020, internalized homophobia was positively associated with depression among those reporting affective orientation towards same-gender persons [48]. A respondent-driven sampling study conducted in 12 Brazilian cities in 2016 observed that moderate or high/very high discrimination due to sexual orientation increased the odds of experiencing moderate/severe depression symptoms [49]. In a longitudinal study from the United States that included young Black and Latino sexual minority men, both racial discrimination and internalized homophobia were associated with depression symptoms [50].

The association between younger age and depressive symptoms among SGD individuals is consistent with previous studies that point to the greater vulnerability of young SGD individuals to mental health issues [51–53]. In the general population, being a young adult can be considered a risk factor for depression, since the transition from adolescence to adulthood is marked by intense changes and can be understood as a crisis in search of a new identity [54]. A systematic review of 42 systematic reviews and meta-analyses reported on the prevalence, severity, and risk factors for mental disorders among SGD persons aged 25 years or lower. Its results showed that SGD youth experienced a heightened risk of depression and a greater severity of symptoms compared to their heterosexual/cisgender peers [55]. In addition, when evaluating suicide attempt, a frequent depression downstream event, another systematic review and meta-analysis of studies published between January 1990 and June 2016 suggested a higher risk of suicide attempts among gay and bisexual persons aged 12 to 26 years compared to their heterosexual counterparts [56].

Our results showing an association between low income and depressive symptoms among *Pardo* and White SGD individuals. Access to basic resources and opportunities have a direct impact on the health status of the population [57]. SGD individuals are often affected by socioeconomic inequalities, lack of access to the labor market, and discrimination in the workplace [58]. The economic inequalities that affect SGD populations are multifaceted and based on gender and sexual discrimination. This may involve the loss of family networks with early leaving or expulsion from home, low learning outcomes and school dropouts, difficulties in accessing and remaining in vocational or undergraduate courses, migration to self-employed or informal jobs, and high rates, especially among trans people, in exploitative sex work [59]. Low income is one of the main barriers to accessing mental health care and, with the scarcity of these services in the public network. As a result, SGD individuals with financial difficulties often do not access prevention services or mental health care [60].

This study has strengths and limitations to be considered when interpreting the results. PHQ-9 is a validated, recognized, and widely used tool to measure depression symptoms, discrimination and internalized homophobia in diverse populations and contexts. In contrast, as the sample is not probabilistic, our findings are not generalizable to a broader population of Brazilian SGD. Due to low sample size, we also could not include in this analysis Indigenous and Asian individuals. We also aggregated all SGM individuals in only one group and did not consider the specificities of each gender identity and sexual orientation. In addition, the analysis accessed individuals who were available to complete the survey, meaning they had internet access and sufficient time. This may have contributed to the greater participation of individuals with higher socioeconomic status. Our primary outcome is a scale-based measure and should not be interpreted as a clinical diagnosis of depression. Finally, as this is a cross-sectional study, it is not possible infer temporality nor causality.

Conclusions

We found a high burden of depressive symptoms among a sample of SGD individuals from Brazil, with higher levels among those self-declaring Black or *Pardo* race. Depressive symptoms were positively associated with discrimination regardless of race. As we advocate for equity, it is crucial to acknowledge and address the unique challenges faced by racial minorities and SGD individuals to inform effective coping strategies. Reducing vulnerability to depression among SGD individuals requires psychosocial approaches that consider the intersecting impacts of racism, homophobia, and transphobia on mental health. Although the PHQ-9 is not a diagnostic tool, the differences found may indicate a gap in access to mental health services and appropriate clinical assessment. Structural interventions must be combined to inclusive, affirming, and equitable mental health services that foster emotional resilience and adaptive coping. Such services are essential to mitigating the

cumulative psychological burden experienced by SGD individuals, who have historically encountered stigma-related barriers to health information, services, and prevention.

Acknowledgements

The authors would like to thank all study participants. This study was presented in part as an e-poster in The AIDS Conference in Munique, Germany, 2024. T.S.T. was supported by Conselho Nacional de Desenvolvimento Científico e Tecnológico (CNPq; #304417/2025-4 and #405558/2025-2. P.M.L. was supported by CNPq (#30700/2025-2) and Fundação Carlos Chagas Filho de Amparo à Pesquisa do Estado do Rio de Janeiro (FAPERJ; #E-26/204.206/2024). B.G. was supported by CNPq (#313265/2023-2) and FAPERJ (#E.26/200.946/2022).

REFERENCES

1. World Health Organization (WHO). Depressive disorder (depression). 26 Aug 2025 [cited 30 Jan 2026]. Available: https://www.who.int/news-room/fact-sheets/detail/depression

2. Evans-Lacko S, Aguilar-Gaxiola S, Al-Hamzawi A, Alonso J, Benjet C, Bruffaerts R, et al. Socio-economic variations in the mental health treatment gap for people with anxiety, mood, and substance use disorders: results from the WHO World Mental Health (WMH) surveys. Psychol Med. 2018;48: 1560–1571. doi:10.1017/S0033291717003336

3. American Psychiatric Association (APA). Manual Diagnóstico e Estatístico de Transtornos Mentais: DSM-5-TR. 5a ed. Porto Alegre, RS, Brasil: Artmed; 2022.

4. Parker R. Interseções entre Estigma, Preconceito e Discriminação na Saúde Pública Mundial. Estigma e saúde. Rio de Janeiro, Brazil: Editora Fiocruz; 2013.

5. Goffman E. Estigma: Notas Sobre a Manipulação da Identidade Deteriorada. 4th ed. Rio de Janeiro, Brazil; 2004.

6. Stuber J, Meyer I, Link B. Stigma, prejudice, discrimination and health. Soc Sci Med. 2008;67: 351–357. doi:10.1016/j.socscimed.2008.03.023

7. Iriart JAB, Castellanos MEP. Preconceito, discriminação e exclusão em saúde. Ciênc Saúde Coletiva. 2023;28: 4–4. doi:10.1590/1413-81232023281.16802022

8. Freitas L, Torres TS, Hoagland B, Silva MST, Veloso VG, Grinsztejn B, et al. Discriminatory experiences among gay, bisexual, and other men who have sex with men, and transgender and non-binary individuals: a cross sectional analysis. Lancet Reg Health - Am. 2024;33: 100737. doi:10.1016/j.lana.2024.100737

9. Lopes F, Werneck J. Saúde da População Negra: da conceituação às políticas públicas de direito. In: WERNECK, J. (Ed.). Mulheres negras: um olhar sobre as lutas sociais e as políticas públicas no Brasil. 1st ed. Mulheres negras: um olhar sobre as lutas sociais e as políticas públicas no Brasil. 1st ed. Rio de Janeiro, Brazil: Criola; 2010.

10. Silva JF, Costa GMC. Health care of sexual and gender minorities: an integrative literature review. Rev Bras Enferm. 2020;73: e20190192. doi:10.1590/0034-7167-2019-0192

11. Ministério da Saúde. Política Nacional de Saúde Integral de Lásbicas, Gays, Bisexuais, Travestis e Transexuais. Brasilia, DF, Brazil; 2013 [cited 30 Jan 2026]. Available: https://bvsms.saude.gov.br/bvs/publicacoes/politica_nacional_saude_lesbicas_gays.pdf

12. Grinshteyn E, Whaley R, Couture M-C. High Fear of Discriminatory Violence among Racial, Gender, and Sexual Minority College Students and Its Association with Anxiety and Depression. Int J Environ Res Public Health. 2022;19: 2117. doi:10.3390/ijerph19042117

13. Werneck J. Racismo institucional e saúde da população negra. Saúde E Soc. 2016;25: 535–549. doi:10.1590/s0104-129020162610

14. Filipa Ferreira A, Caçador Anastácio Z. Perceção da discriminação e saúde mental de jovens e adultos LGBTQI+E não LGBTQI+. Rev INFAD Psicol Int J Dev Educ Psychol. 2022;1: 435–442. doi:10.17060/ijodaep.2022.n1.v1.2401

15. Waller CR, Méndez Leal AS, Silvers JA. Disparities in Depression and Anxiety That Impact Self-Identified Sexual Minority People Affect a Broader Group of Same-Gender Attracted Young Adults. J Adolesc Health. 2023;73: 739–745. doi:10.1016/j.jadohealth.2023.05.024

16. Santos IS, Tavares BF, Munhoz TN, Almeida LSP de, Silva NTB da, Tams BD, et al. Sensibilidade e especificidade do Patient Health Questionnaire-9 (PHQ-9) entre adultos da população geral. Cad Saúde Pública. 2013;29: 1533–1543. doi:10.1590/S0102-311X2013001200006

17. Kroenke K, Spitzer RL, Williams JBW. The PHQ-9: Validity of a brief depression severity measure. J Gen Intern Med. 2001;16: 606–613. doi:10.1046/j.1525-1497.2001.016009606.x

18. Bastos JL, Faerstein E, Celeste RK, Barros AJD. Explicit discrimination and health: development and psychometric properties of an assessment instrument. Rev Saúde Pública. 2012;46: 269–278. doi:10.1590/S0034-89102012000200009

19. Carneiro S. Racismo, sexismo e desigualdade no Brasil. 1st ed. São Paulo, Brazil; 2011.

20. Bernardo FR, Bastos JL, Reichenheim ME. Invariância fatorial da versão abreviada da Escala de Discriminação Explícita em adultos do sul do Brasil. Rev Bras Epidemiol. 2024;27: e240038. doi:10.1590/1980-549720240038.2

21. Bastos JL, Bernardo FR, Reichenheim ME. One step further in mistreatment research: Assessing the scalability of the Explicit Discrimination Scale among Brazilian working-age adult respondents. J Community Psychol. 2025;53: e23146. doi:10.1002/jcop.23146

22. Meyer IH. Prejudice, social stress, and mental health in lesbian, gay, and bisexual populations: Conceptual issues and research evidence. Psychol Bull. 2003;129: 674–697. doi:10.1037/0033-2909.129.5.674

23. Shoptaw S, Weiss RE, Munjas B, Hucks-Ortiz C, Young SD, Larkins S, et al. Homonegativity, Substance Use, Sexual Risk Behaviors, and HIV Status in Poor and Ethnic Men Who Have Sex with Men in Los Angeles. J Urban Health. 2009;86: 77–92. doi:10.1007/s11524-009-9372-5

24. Torres TS, Luz PM, Bezerra DRB, Almeida-Brasil CC, Marins LMS, Veloso VG, et al. Translation and validation in Brazilian Portuguese of the reactions to homosexuality scale. Psychol Health Med. 2021;26: 20–36. doi:10.1080/13548506.2021.1936580

25. Barreto KM, Torres TS, Freitas L, Hoagland B, Jalil EM, Veloso VG, et al. Pre-exposure prophylaxis use and discontinuation among Brazilian gay, bisexual, and other men who have sex with men: examining the role of internalized homonegativity and discrimination. BMC Infect Dis. 2025;25: 1367. doi:10.1186/s12879-025-11742-4

26. Blair KJ, Torres TS, Hoagland B, Bezerra DRB, Veloso VG, Grinsztejn B, et al. Pre-exposure prophylaxis use, HIV knowledge, and internalized homonegativity among men who have sex with men in Brazil: A cross-sectional study. Lancet Reg Health - Am. 2022;6: 100152. doi:10.1016/j.lana.2021.100152

27. Torres TS, Silva MST, Coutinho C, Hoagland B, Jalil EM, Cardoso SW, et al. Evaluation of Mpox Knowledge, Stigma, and Willingness to Vaccinate for Mpox: Cross-Sectional Web-Based Survey Among Sexual and Gender Minorities. JMIR Public Health Surveill. 2023;9: e46489. doi:10.2196/46489

28. Muniz JO, Porto N, Fuks M. GROUPNESS RACIAL E FLUTUAÇÕES ATITUDINAIS DE PARDOS ENTRE FRONTEIRAS SIMBÓLICAS E SOCIAIS. Rev Bras Ciênc Sociais. 2019;34: e3410107. doi:10.1590/3410107/2019

29. Jesus JG. Orientações sobre identidade de gênero: conceitos e termos. Brasilia, DF, Brasil; 2012 [cited 30 Jan 2026]. Available: www.diversidadesexual.com.br/wp-content/uploads/2013/04/GÊNERO-CONCEITOS-E-TERMOS.pdf

30. NIAAA. NIAAA Newsletter. 2004. NIAAA council approves definition of binge drinking. Available: https://pubs.niaaa.nih.gov/publications/ Newsletter/winter2004/Newsletter_Number3.pdf

31. Levis B, Benedetti A, Ioannidis JPA, Sun Y, Negeri Z, He C, et al. Patient Health Questionnaire-9 scores do not accurately estimate depression prevalence: individual participant data meta-analysis. J Clin Epidemiol. 2020;122: 115-128.e1. doi:10.1016/j.jclinepi.2020.02.002

32. Santos IS, Tavares BF, Munhoz TN, Almeida LSPD, Silva NTBD, Tams BD, et al. Sensibilidade e especificidade do Patient Health Questionnaire-9 (PHQ-9) entre adultos da população geral. Cad Saúde Pública. 2013;29: 1533–1543. doi:10.1590/S0102-311X2013001200006

33. Garcia Ferreira AC, Esteves Coelho L, Jalil EM, Luz PM, Friedman RK, Guimarães MRC, et al. Transcendendo: A Cohort Study of HIV-Infected and Uninfected Transgender Women in Rio de Janeiro, Brazil. Transgender Health. 2019;4: 107–117. doi:10.1089/trgh.2018.0063

34. Guimarães MDC, McKinnon K, Dourado I, Veras MA, Magno L, Almeida M, et al. A potential syndemic effect associated with symptoms of depression among men who have sex with men. Braz J Psychiatry. 2022 [cited 30 Jan 2026]. doi:10.47626/1516-4446-2022-2454

35. Instituto Brasileiro de Geografia e Estatística (IBGE). Pesquisa Nacional de Saúde 2019. Brasilia, DF, Brazil; 2020 [cited 30 Jan 2026]. Available: www.pns.icict.fiocruz.br/wp-content/uploads/2021/02/liv101764.pdf

36. Lopes CDS, Gomes NL, Junger WL, Menezes PR. Trend in the prevalence of depressive symptoms in Brazil: results from the Brazilian National Health Survey 2013 and 2019. Cad Saúde Pública. 2022;38: e00123421. doi:10.1590/0102-311x00123421

37. Nascimento A, Silva MF. Impactos do racismo na saúde mental da população negra brasileira. 2021 [cited 30 Jan 2026]. Available: https://periodicosfacesf.com.br/index.php/Psicoatualidades/article/view/271

38. Brito VCDA, Bello-Corassa R, Stopa SR, Sardinha LMV, Dahl CM, Viana MC. Prevalência de depressão autorreferida no Brasil: Pesquisa Nacional de Saúde 2019 e 2013. Epidemiol E Serviços Saúde. 2022;31: e2021384. doi:10.1590/ss2237-9622202200006.especial

39. Damasceno MG, Zanello VML. Saúde Mental e Racismo Contra Negros: Produção Bibliográfica Brasileira dos Últimos Quinze Anos. Psicol Ciênc E Prof. 2018;38: 450–464. doi:10.1590/1982-37030003262017

40. Smolen JR, Araújo EMD. Raça/cor da pele e transtornos mentais no Brasil: uma revisão sistemática. Ciênc Saúde Coletiva. 2017;22: 4021–4030. doi:10.1590/1413-812320172212.19782016

41. Luz PM, Jalil EM, Castilho J, Velasque L, Ramos M, Ferreira ACG, et al. Association of Discrimination, Violence, and Resilience with Depressive Symptoms Among Transgender Women in Rio de Janeiro, Brazil: A Cross-Sectional Analysis. Transgender Health. 2022;7: 101–106. doi:10.1089/trgh.2020.0171

42. Dalgalarrondo P. Psicopatologia e Semiologia dos Transtornos Mentais. 3rd ed. Porto Alegre, RS, Brazil: Artmed; 2019. Available: https://membros.analysispsicologia.com.br/wp-content/uploads/2024/06/Psicopatologia-Paulo-Dalgalarrondo.pdf

43. Veiga LM. Descolonizando a psicologia: notas para uma Psicologia Preta. Fractal Rev Psicol. 2019;31: 244. doi:10.22409/1984-0292/v31i_esp/29000

44. Zamora MHRN. Desigualdade racial, racismo e seus efeitos. Fractal Rev Psicol. 2012;24: 563–578. doi:10.1590/S1984-02922012000300009

45. Tavares JSC, Kuratani SMDA. Manejo Clínico das Repercussões do Racismo entre Mulheres que se “Tornaram Negras.” Psicol Ciênc E Prof. 2019;39: e184764. doi:10.1590/1982-3703003184764

46. Nicácio JDJ. O racismo antinegro, a psicanálise e a subjetividade da nossa época no Brasil. Afro-Ásia. 2020 [cited 30 Jan 2026]. doi:10.9771/aa.v0i62.42891

47. Silva RLD, Souza LLD. Temporalidades e racialização de jovens gays negros: Entre memórias e imagens de futuro. Sex Salud Soc Rio Jan. 2023; e22317. doi:10.1590/1984-6487.sess.2023.39.e22317.a.pt

48. Batista TS, Tavares FMDP, Gonçalves GP, Torres JL. Homofobia internalizada e depressão em mulheres e homens homossexuais e bissexuais: Inquérito de saúde LGBT+, 2020. Ciênc Saúde Coletiva. 2024;29: e05412023. doi:10.1590/1413-81232024299.05412023

49. Guimarães MDC, McKinnon K, Dourado I, Veras MA, Magno L, Almeida M, et al. A potential syndemic effect associated with symptoms of depression among men who have sex with men. Braz J Psychiatry. 2022 [cited 30 Jan 2026]. doi:10.47626/1516-4446-2022-2454

50. DiGuiseppi GT, Davis JP, Srivastava A, Layland EK, Pham D, Kipke MD. Multiple Minority Stress and Behavioral Health Among Young Black and Latino Sexual Minority Men. LGBT Health. 2022;9: 114–121. doi:10.1089/lgbt.2021.0230

51. Guz S, Kattari SK, Atteberry-Ash B, Klemmer CL, Call J, Kattari L. Depression and Suicide Risk at the Cross-Section of Sexual Orientation and Gender Identity for Youth. J Adolesc Health. 2021;68: 317–323. doi:10.1016/j.jadohealth.2020.06.008

52. Helminen EC, Scheer JR, Ash TL, Haik AK, Felver JC. Discrimination, Depression, and Anxiety Among Sexual Minority and Heterosexual Young Adults: The Role of Self-Compassion. LGBT Health. 2023;10: 315–323. doi:10.1089/lgbt.2022.0079

53. Simões JA, França IL, Macedo M. Jeitos de corpo: cor/raça, gênero, sexualidade e sociabilidade juvenil no centro de São Paulo. Cad Pagu. 2010; 37–78. doi:10.1590/S0104-83332010000200003

54. Lopez MRA, Ribeiro JP, Ores LDC, Jansen K, Souza LDDM, Pinheiro RT, et al. Depressão e qualidade de vida em jovens de 18 a 24 anos no sul do Brasil. Rev Psiquiatr Rio Gd Sul. 2011;33: 103–108. doi:10.1590/S0101-81082011005000001

55. O'Shea J, Jenkins R, Nicholls D, Downs J, Hudson LD. Prevalence, severity and risk factors for mental disorders among sexual and gender minority young people: a systematic review of systematic reviews and meta-analyses. Eur Child Adolesc Psychiatry. 2025;34: 959–982. doi:10.1007/s00787-024-02552-1

56. Miranda-Mendizábal A, Castellví P, Parés-Badell O, Almenara J, Alonso I, Blasco MJ, et al. Sexual orientation and suicidal behaviour in adolescents and young adults: systematic review and meta-analysis. Br J Psychiatry. 2017;211: 77–87. doi:10.1192/bjp.bp.116.196345

57. Mello L, Avelar RBD, Maroja D. Por onde andam as políticas públicas para a população LGBT no Brasil. Soc E Estado. 2012;27: 289–312. doi:10.1590/S0102-69922012000200005

58. Suliano D, Irffi G, Rêgo De Sá Barreto AB. Orientação sexual e seus efeitos no mercado de trabalho: um estudo com base na técnica de revisão sistemática. Rev Bras Estud Popul. 2022;39: 1–29. doi:10.20947/S0102-3098a0186

59. Instituto Matizes. Inclusão econômica e geração de renda da população LGBTQIA+ no Brasil: Desafios, iniciativas e financiamentos. São Paulo, Brazil: Instituto Matizes; 2024 [cited 30 Jan 2026]. Available: https://fundopositivo.org.br/wp-content/uploads/2024/08/Inclusao-relatorio_digital_05-1.pdf

60. Cardoso MR, Ferro LF. Saúde e população LGBT: demandas e especificidades em questão. Psicol Ciênc E Prof. 2012;32: 552–563. doi:10.1590/S1414-98932012000300003

Table 1. Sociodemographic characteristics, HIV status, discrimination and internalized homophobia overall and stratified by race.

	Total N=6220	White N=3749 (60.3%)	*Pardo* N=1685 (21.3%)	Black N=786 (12.6%)	p-value
**Recruitment**					< 0.001
Grindr	1313 (21.1)	742 (19.8)	387 (23.0)	184 (23.4)	
Hornet	2386 (38.4)	1563 (41.7)	578 (34.3)	245 (31.2)	
Scruff	1939 (31.2)	1117 (29.8)	545 (32.3)	277 (35.2)	
Facebook/Instagram	582 (9.4)	327 (8.7)	175 (10.4)	80 (10.2)	
**Age (years)**					
Median (IQR)	36 (29-44)	37 (30-45)	35 (29-42)	33 (27-39)	< 0.001
18-24	543 (8.7)	292 (7.8)	160 (9.5)	91 (11.6)	< 0.001
25-35	2514 (40.4)	1406 (37.5)	722 (42.8)	386 (49.1)	
>35	3163 (50.9)	2051 (54.7)	803 (47.7)	309 (39.3)	
**Gender**					0.005
Cisgender man	6081 (97.8)	3670 (97.9)	1653 (98.1)	758 (96.4)	
Transgender man	12 (0.2)	4 (0.1)	5 (0.3)	3 (0.4)	
Transgender woman	12 (0.2)	6 (0.2)	2 (0.1)	4 (0.5)	
Travesti	5 (0.1)	1 (0)	4 (0.2)	0 (0)	
Non-binary person	110 (1.8)	68 (1.8)	21 (1.2)	21 (2.7)	
**Sexual orientation**					< 0.001
Gay	5226 (84.1)	3236 (86.4)	1372 (81.5)	618 (78.8)	
Bisexual	803 (12.9)	415 (11.1)	262 (15.6)	126 (16.1)	
Heterosexual	63 (1.0)	36 (1.0)	18 (1.1)	9 (1.1)	
Other	121 (1.9)	59 (1.6)	31 (1.8)	31 (4.0)	
Missing[a]	7 (0.0)	3 (0.0)	2 (0.0)	2 (0.0)	
**Education**					< 0.001
Elementary or less	136 (2.2)	73 (1.9)	48 (2.8)	15 (1.9)	
Secondary	1365 (21.9)	684 (18.2)	448 (26.6)	233 (29.6)	
Terciary or higher	4692 (75.4)	2981 (79.5)	1180 (70.0)	531 (67.6)	
Preferred not to answer	27 (0.4)	11 (0.3)	9 (0.5)	7 (0.9)	
**Monthly family income**[1]					< 0.001
Low	1393 (22.4)	638 (17.0)	500 (29.7)	255 (32.4)	
Middle	2740 (44.1)	1621 (43.2)	752 (44.6)	367 (46.7)	
High	1872 (30.1)	1338 (35.7)	387 (23.0)	147 (18.7)	
Preferred not to answer	215 (3.5)	152 (4.1)	46 (2.7)	17 (2.2)	
**Country region**					< 0.001
North	100 (1.6)	46 (1.2)	47 (2.8)	7 (0.9)	
North East	717 (11.5)	275 (7.3)	306 (18.2)	136 (17.3)	
Midwest	392 (6.3)	201 (5.4)	143 (8.5)	48 (6.1)	
Southeast	4300 (69.1)	2702 (72.1)	1055 (62.6)	543 (69.1)	
South	711 (11.4)	525 (14.0)	134 (8.0)	52 (6.6)	
**Living in a state capital**					< 0.001
No	1657 (26.6)	1082 (28.9)	408 (24.2)	167 (21.2)	
Yes	4563 (73.4)	2667 (71.1)	1277 (75.8)	619 (78.8)	
**Illicit substance use**					0.013
No	4001 (64.3)	2368 (63.2)	1133 (67.2)	500 (63.6)	
Yes	2219 (35.7)	1381 (36.8)	552 (32.8)	286 (36.4)	
**Binge drinking**					<0.001

No	4351 (70.0)	2716 (72.4)	1147 (68.1)	488 (62.1)	
Yes	1869 (30.0)	1033 (27.6)	538 (31.9)	298 (37.9)	
**HIV status**					0.68
Negative	4221 (67.9)	2567 (68.5)	1123 (66.7)	531 (67.6)	
Positive	1590 (25.6)	934 (24.9)	450 (26.7)	206 (26.2)	
Unknown	407 (6.5)	247 (6.6)	111 (6.6)	49 (6.2)	
Missing[a]	2 (0.0)	1 (0.0)	1 (0.0)	0 (0)	
**Internalized homophobia scores**[2] **(mean; SD)**	10.3 (8.2)	10.1 (8.2)	10.9 (8.3)	9.6 (8.0)	< 0.001
**Discrimination scores**[3] **(mean; SD)**	3.5 (3.8)	3.1 (3.4)	3.7 (3.9)	5.2 (4.6)	< 0.001
**Depressive symptoms (PHQ-9)** [4]					
Mean scores (SD)	8.8 (7.1)	8.4 (7.0)	9.3 (7.2)	9.6 (7.5)	<0.001
No symptoms	2030 (33.2)	1296 (35.1)	501 (30.4)	233 (30.3)	<0.001
Mild symptoms	1961 (32.1)	1203 (32.6)	518 (31.5)	240 (31.2)	
Moderate symptoms	882 (14.4)	512 (13.9)	249 (15.1)	121 (15.7)	
Moderate to severe symptoms	647 (10.6)	354 (9.6)	215 (13.1)	78 (10.1)	
Severe symptoms	587 (9.6)	326 (8.8)	163 (9.9)	98 (12.7)	

IQR: interquartile range, SD: standard deviation

[1] Monthly family income based on the minimum wage (R$1,212.00 in 2021, equivalent to US$242.00)

[2] Internalized Homophobia Scale

[3] Explicit Discrimination Scale

[4] No symptoms (0-4 points); mild symptoms (5-9 points); moderate symptoms (10-14 points); moderate to severe symptoms (15-19 points); severe symptoms (20-27 points)

[a] *Missing*: missing information provided for all variables that had missing data

Table 2. Reported reasons for discrimination across the 8-items of the Explicit Discrimination Scale stratified by race.

	**White (N,%)**	**Pardo (N,%)**	**Black (N,%)**
**Total**	3749 (100.0)	1685 (100.0)	786 (100.0)
Race	97 (2.6)	548 (32.5)	612 (77.9)
Social class	928 (24.8)	706 (41.9)	415 (52.8)
Sexual orientation	2366 (63.1)	1043 (61.9)	498 (63.4)
Gender	540 (14.4)	268 (15.9)	131 (16.7)
Disease	87 (2.3)	44 (2.6)	12 (1.5)
Age	458 (12.2)	247 (14.7)	129 (16.4)
Housing location	251 (6.7)	216 (12.8)	130 (16.5)
Accent	498 (13.3)	300 (17.8)	114 (14.5)
Clothing	51 (1.4)	14 (0.8)	2 (0.3)
Overweight	550 (14.7)	244 (14.5)	103 (13.1)
Disability	49 (1.3)	30 (1.8)	14 (1.8)
Appearance	66 (1.8)	28 (1.7)	4 (0.5)
Political beliefs	433 (11.5)	189 (11.2)	99 (12.6)
Religion	224 (6.0)	136 (8.1)	85 (10.8)
Other	133 (3.5)	39 (2.3)	14 (1.8)

Table 3. Linear regression models between discrimination, internalized homophobia, and other co-variables with depressive symptoms, stratified by race.

	**White**		***Pardo***		**Black**	
	Estimate (SD)	p-value	Estimate (SD)	p-value	Estimate (SD)	p-value
Univariable models of main exposures						
**Discrimination scores[2]**	**0.40 (0.02)**	**<0.0001**	**0.36 (0.02)**	**<0.0001**	**0.32 (0.03)**	**<0.0001**
**Internalized homophobia scores[3]**	**0.03 (0.02)**	**0.045**	**0.05 (0.02)**	**0.035**	-0.01 (0.04)	0.72
Multivariable model (main exposures combined)						
**Discrimination scores**	**0.39 (0.02)**	**<0.0001**	**0.36 (0.02)**	**<0.0001**	**0.32 (0.03)**	**<0.0001**
**Internalized homophobia scores**	**0.04 (0.01)**	**0.0072**	**0.05 (0.02)**	**0.030**	-0.01 (0.04)	0.71
Multivariable model (main exposures and co-variables combined)						
**Discrimination scores**	**0.36 (0.02)**	**<0.0001**	**0.34 (0.03)**	**<0.0001**	**0.30 (0.03)**	**<0.0001**
**Internalized homophobia scores**	**0.08 (0.02)**	**<0.0001**	**0.08 (0.02)**	**0.0011**	0.01 (0.04)	0.71
**Age (per 10-year increase)**	**-0.18 (0.01)**	**<0.0001**	**-0.21 (0.03)**	**<0.0001**	**-0.24 (0.04)**	**<0.0001**
**Gender**						
Cisgender man	Ref.		Ref.		Ref.	
Trans or non-binary person	1.19 (0.12)	0.10	0.04 (020)	0.84	0.37 (0.23)	0.10
**Sexual orientation**						
Gay	Ref.		Ref.		Ref.	
Other	0.01 (0.05)	0.91	0.09 (0.07)	0.76	-0.05 (0.10)	0.62
**Education**						
Elementary or less	-0.02 (0.12)	0.87	-0.01 (0.15)	0.92	0.02 (0.26)	0.94
Secondary	0.04 (0.04)	0.28	0.02 (0.06)	0.76	0.10 (0.08)	0.26
Terciary	Ref.		Ref.		Ref.	
**Monthly family income[1]**						
Low	**0.19 (0.05)**	**<0.0001**	**0.29 (0.07)**	**<0.0001**	0.15 (0.11)	0.18
Middle	**0.11 (0.03)**	**<0.0001**	0.07 (0.06)	0.24	-0.01 (0.10)	0.90
High	Ref.		Ref.		Ref.	
**Region**						
North, Northeast or Midwest	-0.03 (0.04)	0.55	-0.03 (0.05)	0.52	0.06 (0.08)	0.45
Southeast or South	Ref.		Ref.		Ref.	
**Lives in a state capital**						
No	0.01 (0.03)	0.84	-0.03 (0.05)	0.56	0.02 (0.09)	0.77
Yes	Ref.		Ref.		Ref.	
**Illicit substance use**						
No	Ref.		Ref.		Ref.	
Yes	0.01 (0.03)	0.69	0.06 (0.05)	0.27	0.14 (0.08)	0.065
**Binge drinking**						
No	**Ref.**		Ref.		Ref.	
Yes	**0.08 (0.04)**	**0.026**	0.09 (0.05)	0.076	0.05 (0.08)	0.51
**HIV status**						
Negative	Ref.		Ref.		Ref.	
Positive	0.01 (0.04)	0.89	0.03 (0.05)	0.56	0.03 (0.08)	0.74
Unknown	-0.01 (0.06)	0.89	-0.03 (0.10)	0.74	-0.08 (0.15)	0.60

Depressive symptoms accessed with PHQ-9 scores. SD: standard deviation. **Bold**: $p<0.05$; [1] Monthly family income based on the minimum wage (MW) which was R$1,212.00 in 2021 (US$242.00); [2] Internalized Homophobia Scale; [3] Explicit Discrimination Scale

Figure 1. Study flowchart, Brazil, November 2021 to January 2022.

Figure 2: Percentage of individuals reporting frequency of symptoms for each PHQ-9 item according to race, Brazil, November 2021 to January 2022.

Legend: 0: not at all; 1: several days; 2: more than half the days; 3: almost every day

Figure 1 Click here to access/download;Figure;figure1.png

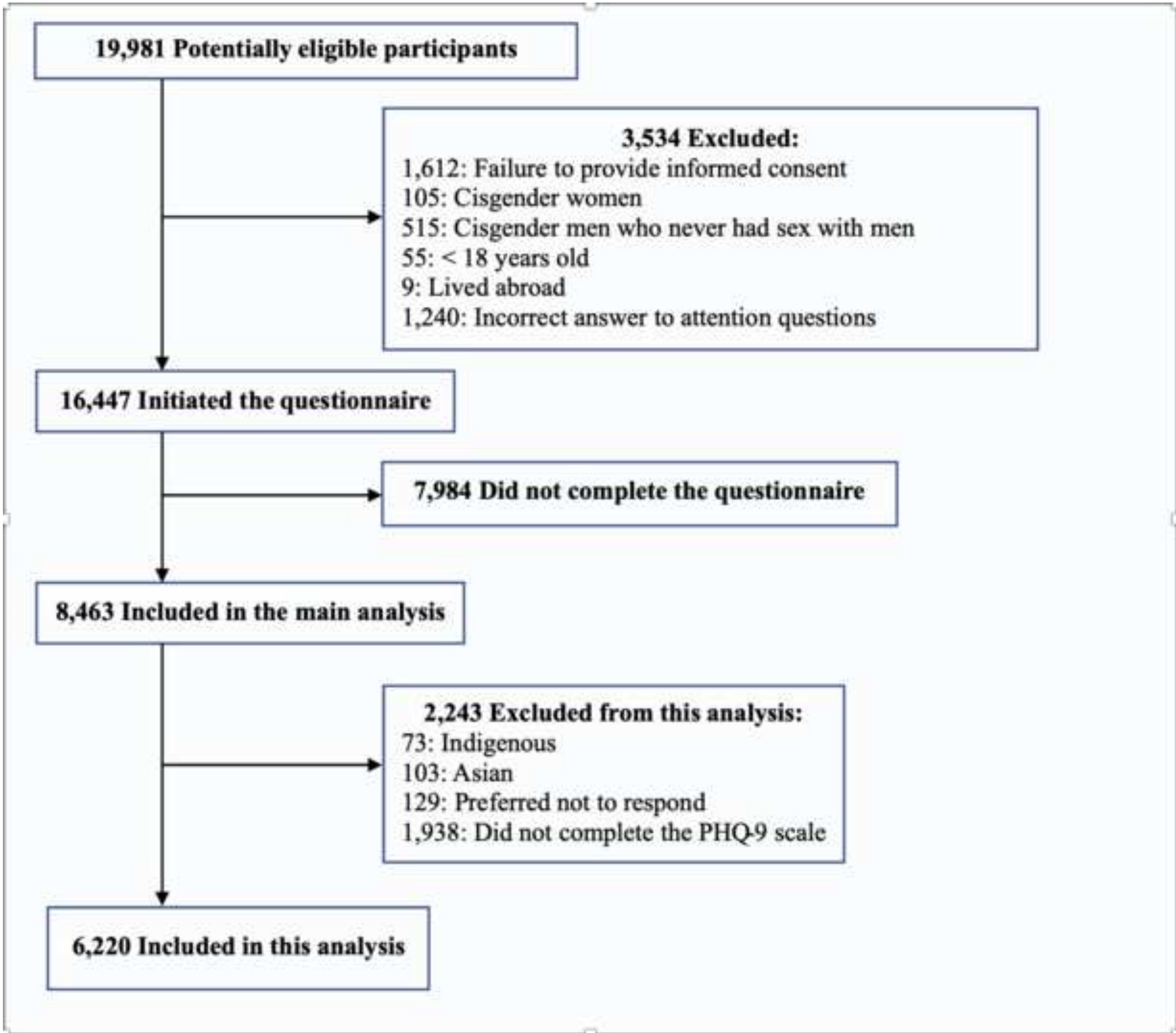

Figure 2

Click here to access/download;Figure;figure2.png

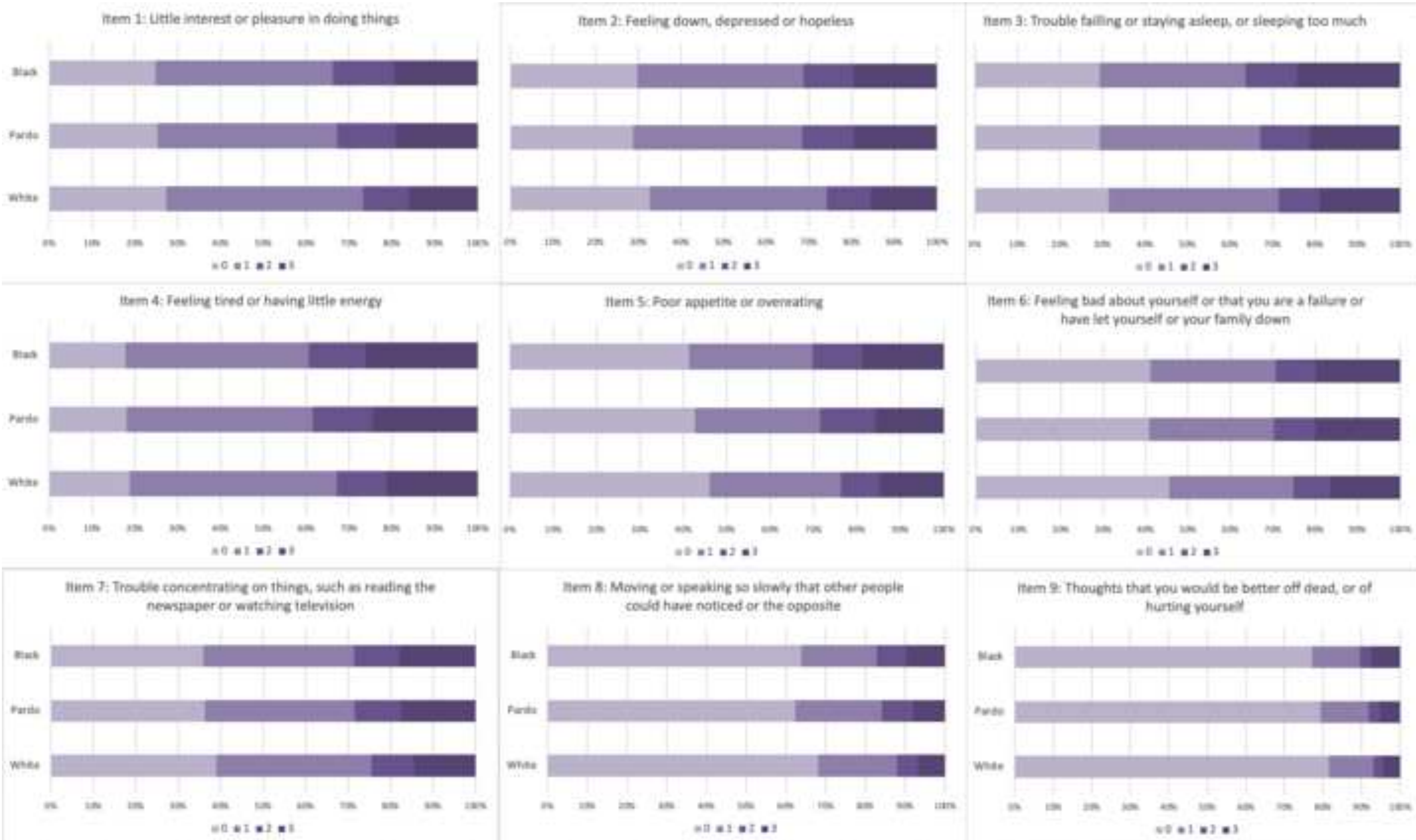

Supplement: Supplementary file 1 [file pgph.0007276.s002.pdf]
