# Supplementary material for: High burden of depressive symptoms among sexual and gender diverse persons in Brazil: The role of discrimination, internalized homonegativity, and race
Source: PLOS Glob Public Health. 2026 Sep 28;6(9):e0007276. doi: 10.1371/journal.pgph.0007276 (PMC13618917; doi:10.1371/journal.pgph.0007276)
Supplement: Supplementary file 3 [file pgph.0007276.s004.docx]

Academic Editor Comments:
The manuscript uses both sexual and gender diverse (SGD) and LGBTQIAPN+ terminology. I suggest standardising the terminology throughout the manuscript, where appropriate, by using either SGD or LGBTQIAPN+. An exception may be warranted when describing or citing previous research, in which case the terminology used by the original authors may be retained to accurately reflect the population studied. Additionally, LGBTQIAPN+ appears to be introduced for the first time in the Methods section. Please consider defining the acronym in full when it is first introduced, preferably in the Introduction.

Response: We decided to keep SGD throughout the manuscript. An exception is the description of the RHS scale in the methods, as we are citing previous research.

References 19 and 34 appear to be duplicates. Please review the reference list and remove the duplicate if appropriate: Santos IS, Tavares BF, Munhoz TN, Almeida LSP de, Silva NTB da, Tams BD, et al. Sensibilidade e especificidade do Patient Health Questionnaire-9 (PHQ-9) entre adultos da população geral. Cad Saúde Pública. 2013;29:1533–1543. doi:10.1590/S0102-311X2013001200006.

Response: We removed the duplicated reference.

Reviewer 1
This is a re-review and the authors have addressed all comments.

Comments: Thanks

Reviewer 2
The authors have satisfactorily addressed the comments raised by the reviewers. The revised manuscript is clearer, better structured, and now aligns with PLOS publication guidance. The topic is important and timely, and the manuscript makes a meaningful contribution to the field.

The manuscript is written in clear English, and the statistical analysis appears appropriate for the study aims and data presented. The revisions have strengthened the clarity of the methods, results, and interpretation of findings. I do not have any remaining major concerns regarding the manuscript.

I also do not identify concerns related to dual publication, research ethics, or publication ethics based on the revised submission. Overall, I believe the manuscript is suitable for publication.

Comments: Thanks
