# Supplementary material for: High burden of depressive symptoms among sexual and gender diverse persons in Brazil: The role of discrimination, internalized homonegativity, and race
Source: PLOS Glob Public Health. 2026 Sep 28;6(9):e0007276. doi: 10.1371/journal.pgph.0007276 (PMC13618917; doi:10.1371/journal.pgph.0007276)
Supplement: Peer review history

# Peer review: Decision Letter 0

**DOI:** 10.1371/journal.pgph.0007276.r001

**Authors:** Jen Edwards

22 Mar 2026

PGPH-D-26-00431

High burden of depressive symptoms among sexual and gender diverse individuals of Black race in Brazil: the role of discrimination and internalized homophobia

PLOS Global Public Health

Dear Dr. Torres,

Thank you for submitting your manuscript to PLOS Global Public Health. After careful consideration, we feel that it has merit but does not fully meet PLOS Global Public Health’s publication criteria as it currently stands. Therefore, we invite you to submit a revised version of the manuscript that addresses the points raised during the review process.

Please note that we have only been able to secure a single reviewer to assess your manuscript. We are issuing a decision on your manuscript at this point to prevent further delays in the evaluation of your manuscript. Please be aware that the editor who handles your revised manuscript might find it necessary to invite additional reviewers to assess this work once the revised manuscript is submitted. However, we will aim to proceed on the basis of this single review if possible.

The reviewer raised a number of points which need to be addressed, including the need for clearer discussion of the study limitations and theoretical frameworks. Could you please carefully revise the manuscript to address all comments raised?

Please submit your revised manuscript by May 04 2026 11:59PM. If you will need more time than this to complete your revisions, please reply to this message or contact the journal office at globalpubhealth@plos.org. When you're ready to submit your revision, log on to https://www.editorialmanager.com/pgph/ and select the 'Submissions Needing Revision' folder to locate your manuscript file.

Please include the following items when submitting your revised manuscript:

- A letter that responds to each point raised by the editor and reviewer(s). You should upload this letter as a separate file labeled 'Response to Reviewers'.
- A marked-up copy of your manuscript that highlights changes made to the original version. You should upload this as a separate file labeled 'Revised Manuscript with Track Changes'.
- An unmarked version of your revised paper without tracked changes. You should upload this as a separate file labeled 'Manuscript'.

Guidelines for resubmitting your figure files are available below the reviewer comments at the end of this letter.

We look forward to receiving your revised manuscript.

Kind regards,

Jen Edwards

Staff Editor

PLOS Global Public Health

Journal Requirements:

1. Please provide separate figure files in .tif or .eps format.

For more information about figure files please see our guidelines:  LINK

https://journals.plos.org/globalpublichealth/s/figures

https://journals.plos.org/globalpublichealth/s/figures#loc-file-requirements

If the reviewer comments include a recommendation to cite specific previously published works, please review and evaluate these publications to determine whether they are relevant and should be cited. There is no requirement to cite these works unless the editor has indicated otherwise.

Additional Editor Comments (if provided):

[Note: HTML markup is below. Please do not edit.]

Reviewers' comments:

Reviewer’s Responses to Questions

**Comments to the Author**

1. Does this manuscript meet PLOS Global Public Health’s publication criteria?

Reviewer #1: Yes

**********

2. Has the statistical analysis been performed appropriately and rigorously?

Reviewer #1: Yes

**********

3. Have the authors made all data underlying the findings in their manuscript fully available (please refer to the Data Availability Statement at the start of the manuscript PDF file)?

The PLOS Data policy

Reviewer #1: Yes

**********

4. Is the manuscript presented in an intelligible fashion and written in standard English?

Reviewer #1: Yes

**********

Reviewer #1: he study addresses an important public health issue and contributes to the literature on mental health disparities among marginalized populations in the Global South. A key strength of the paper is its large sample size and the use of validated measures, as well as the stratification of analyses by race, which provides insight into racial differences in mental health outcomes within sexual and gender diverse populations. The findings highlight a high burden of depressive symptoms, particularly among Black participants, and demonstrate a consistent relationship between experiences of discrimination and poorer mental health.

However, the manuscript would benefit from stronger integration of theoretical frameworks such as minority stress and intersectionality, clearer discussion of methodological limitations related to the convenience sampling approach, and greater attention to language clarity and consistency. With these improvements, the paper has the potential to make a meaningful contribution to research on sexual and gender minority health and the role of discrimination in shaping mental health outcomes.

Lastly, I have included some comments and recommendation on the manuscript.

**********

what does this mean?). If published, this will include your full peer review and any attached files.

**Do you want your identity to be public for this peer review?**  If you choose “no”, your identity will remain anonymous but your review may still be made public.

For information about this choice, including consent withdrawal, please see our Privacy Policy

Reviewer #1: **Yes:** Lebogang Manthibe Ramalepe

**********

[NOTE: If reviewer comments were submitted as an attachment file, they will be attached to this email and accessible via the submission site. Please log into your account, locate the manuscript record, and check for the action link "View Attachments". If this link does not appear, there are no attachment files.]

Attachment Submitted filename: PGPH-D-26-00431.pdf

# Author comment: Author response to Decision Letter 1

**DOI:** 10.1371/journal.pgph.0007276.r002

8 Apr 2026

Attachment Submitted filename: PGPH-D-26-00431 (3).pdf

# Peer review: Decision Letter 1

**DOI:** 10.1371/journal.pgph.0007276.r003

**Authors:** Matthew N Berger

19 Aug 2026

PGPH-D-26-00431R1

High burden of depressive symptoms among sexual and gender diverse persons in Brazil: the role of discrimination, internalized homonegativity, and race

PLOS Global Public Health

Dear Dr. Torres,

Thank you for submitting your manuscript to PLOS Global Public Health. After careful consideration, we feel that it has merit but does not fully meet PLOS Global Public Health’s publication criteria as it currently stands. Therefore, we invite you to submit a revised version of the manuscript that addresses the points raised during the review process.

Please submit your revised manuscript by Sep 18 2026 11:59PM. If you will need more time than this to complete your revisions, please reply to this message or contact the journal office at globalpubhealth@plos.org. When you're ready to submit your revision, log on to https://www.editorialmanager.com/pgph/ and select the 'Submissions Needing Revision' folder to locate your manuscript file.

Please include the following items when submitting your revised manuscript:

- A letter that responds to each point raised by the editor and reviewer(s). You should upload this letter as a separate file labeled 'Response to Reviewers'.
- A marked-up copy of your manuscript that highlights changes made to the original version. You should upload this as a separate file labeled 'Revised Manuscript with Track Changes'.
- An unmarked version of your revised paper without tracked changes. You should upload this as a separate file labeled 'Manuscript'.

Guidelines for resubmitting your figure files are available below the reviewer comments at the end of this letter.

As the corresponding author, your ORCID iD is verified in the submission system and will appear in the published article. PLOS supports the use of ORCID, and we encourage all coauthors to register for an ORCID iD and use it as well. Please encourage your coauthors to verify their ORCID iD within the submission system before final acceptance, as unverified ORCID iDs will not appear in the published article. *Only*  the individual author can complete the verification step; PLOS staff *cannot*  verify ORCID iDs on behalf of authors.

We look forward to receiving your revised manuscript.

Kind regards,

Matthew N Berger, PhD

Academic Editor

PLOS Global Public Health

**Journal Requirements:**

If the reviewer comments include a recommendation to cite specific previously published works, please review and evaluate these publications to determine whether they are relevant and should be cited. There is no requirement to cite these works unless the editor has indicated otherwise.

Please review your reference list to ensure that it is complete and correct. If you have cited papers that have been retracted, please include the rationale for doing so in the manuscript text, or remove these references and replace them with relevant current references. Any changes to the reference list should be mentioned in the rebuttal letter that accompanies your revised manuscript. If you need to cite a retracted article, indicate the article’s retracted status in the References list and also include a citation and full reference for the retraction notice.

**Additional Editor Comments (if provided):**

Academic Editor Comments:

The manuscript uses both sexual and gender diverse (SGD) and LGBTQIAPN+ terminology. I suggest standardising the terminology throughout the manuscript, where appropriate, by using either SGD or LGBTQIAPN+. An exception may be warranted when describing or citing previous research, in which case the terminology used by the original authors may be retained to accurately reflect the population studied. Additionally, LGBTQIAPN+ appears to be introduced for the first time in the Methods section. Please consider defining the acronym in full when it is first introduced, preferably in the Introduction.

References 19 and 34 appear to be duplicates. Please review the reference list and remove the duplicate if appropriate: Santos IS, Tavares BF, Munhoz TN, Almeida LSP de, Silva NTB da, Tams BD, et al. Sensibilidade e especificidade do Patient Health Questionnaire-9 (PHQ-9) entre adultos da população geral. Cad Saúde Pública. 2013;29:1533–1543. doi:10.1590/S0102-311X2013001200006.

Reviewer 1

This is a re-review and the authors have addressed all comments.

Reviewer 2

The authors have satisfactorily addressed the comments raised by the reviewers. The revised manuscript is clearer, better structured, and now aligns with PLOS publication guidance. The topic is important and timely, and the manuscript makes a meaningful contribution to the field.

The manuscript is written in clear English, and the statistical analysis appears appropriate for the study aims and data presented. The revisions have strengthened the clarity of the methods, results, and interpretation of findings. I do not have any remaining major concerns regarding the manuscript.

I also do not identify concerns related to dual publication, research ethics, or publication ethics based on the revised submission. Overall, I believe the manuscript is suitable for publication.

[Note: HTML markup is below. Please do not edit.]

Reviewers' comments:

Reviewer’s Responses to Questions

**Comments to the Author**

Reviewer #1: All comments have been addressed

Reviewer #2: All comments have been addressed

**********

publication criteria?

Reviewer #1: Yes

Reviewer #2: Yes

**********

3. Has the statistical analysis been performed appropriately and rigorously?

Reviewer #1: Yes

Reviewer #2: Yes

**********

4. Have the authors made all data underlying the findings in their manuscript fully available (please refer to the Data Availability Statement at the start of the manuscript PDF file)?

The PLOS Data policy

Reviewer #1: Yes

Reviewer #2: Yes

**********

5. Is the manuscript presented in an intelligible fashion and written in standard English?

Reviewer #1: Yes

Reviewer #2: Yes

**********

**Reviewer #1:**  This is a re-review and the authors have addressed all comments.

**Reviewer #2:** The authors have satisfactorily addressed the comments raised by the reviewers. The revised manuscript is clearer, better structured, and now aligns with PLOS publication guidance. The topic is important and timely, and the manuscript makes a meaningful contribution to the field.

The manuscript is written in clear English, and the statistical analysis appears appropriate for the study aims and data presented. The revisions have strengthened the clarity of the methods, results, and interpretation of findings. I do not have any remaining major concerns regarding the manuscript.

I also do not identify concerns related to dual publication, research ethics, or publication ethics based on the revised submission. Overall, I believe the manuscript is suitable for publication.

**********

what does this mean?). If published, this will include your full peer review and any attached files.

**Do you want your identity to be public for this peer review?**  If you choose “no”, your identity will remain anonymous but your review may still be made public.

For information about this choice, including consent withdrawal, please see our Privacy Policy

Reviewer #1: **Yes:** Lebogang Mathibe Ramalepe

Reviewer #2: No

**********

[NOTE: If reviewer comments were submitted as an attachment file, they will be attached to this email and accessible via the submission site. Please log into your account, locate the manuscript record, and check for the action link "View Attachments". If this link does not appear, there are no attachment files.]

**Figure Resubmissions:**

# Author comment: Author response to Decision Letter 2

**DOI:** 10.1371/journal.pgph.0007276.r004

19 Aug 2026

Attachment Submitted filename: reply2.docx

# Editor report: Decision Letter 2

**DOI:** 10.1371/journal.pgph.0007276.r005

**Authors:** Matthew N Berger

23 Aug 2026

High burden of depressive symptoms among sexual and gender diverse persons in Brazil: the role of discrimination, internalized homonegativity, and race

PGPH-D-26-00431R2

Dear Dr. Torres,

We are pleased to inform you that your manuscript 'High burden of depressive symptoms among sexual and gender diverse persons in Brazil: the role of discrimination, internalized homonegativity, and race' has been provisionally accepted for publication in PLOS Global Public Health.

Before your manuscript can be formally accepted you will need to complete some formatting changes, which you will receive in a follow up email. A member of our team will be in touch with a set of requests.

Please note that your manuscript will not be scheduled for publication until you have made the required changes, so a swift response is appreciated.

IMPORTANT: The editorial review process is now complete. PLOS will only permit corrections to spelling, formatting or significant scientific errors from this point onwards. Requests for major changes, or any which affect the scientific understanding of your work, will cause delays to the publication date of your manuscript.

If your institution or institutions have a press office, please notify them about your upcoming paper to help maximize its impact. If they'll be preparing press materials, please inform our press team as soon as possible -- no later than 48 hours after receiving the formal acceptance. Your manuscript will remain under strict press embargo until 2 pm Eastern Time on the date of publication. For more information, please contact globalpubhealth@plos.org.

Thank you again for supporting Open Access publishing; we are looking forward to publishing your work in PLOS Global Public Health.

Best regards,

Matthew N Berger, PhD

Academic Editor

PLOS Global Public Health

***********************************************************

Reviewer Comments (if any, and for reference):
